# Ecophysiology of the cosmopolitan nitrite-oxidizing genus *Candidatus* Nitromaricola

**DOI:** 10.1093/ismejo/wrag271

**Published:** 2026-10-19

**Authors:** Joerdis Stuehrenberg, Jan N von Arx, Katharina Kitzinger, Gaute Lavik, Sten Littmann, Hannah K Marchant, Jana Milucka, Luis H Orellana, Marcel M M Kuypers

**Affiliations:** Max Planck Institute for Marine Microbiology, Celsiusstrasse 1, 28359 Bremen, Germany; Max Planck Institute for Marine Microbiology, Celsiusstrasse 1, 28359 Bremen, Germany; Max Planck Institute for Marine Microbiology, Celsiusstrasse 1, 28359 Bremen, Germany; Max Planck Institute for Marine Microbiology, Celsiusstrasse 1, 28359 Bremen, Germany; Max Planck Institute for Marine Microbiology, Celsiusstrasse 1, 28359 Bremen, Germany; Max Planck Institute for Marine Microbiology, Celsiusstrasse 1, 28359 Bremen, Germany; MARUM - Centre for Marine Environmental Sciences University of Bremen, Leobener Strasse 8, 28359 Bremen, Germany; Max Planck Institute for Marine Microbiology, Celsiusstrasse 1, 28359 Bremen, Germany; Max Planck Institute for Marine Microbiology, Celsiusstrasse 1, 28359 Bremen, Germany; Max Planck Institute for Marine Microbiology, Celsiusstrasse 1, 28359 Bremen, Germany

**Keywords:** nitrite oxidation, nitrite-oxidizing bacteria, biogeochemistry, environmental microbiology, physiology, Black Sea

## Abstract

The majority of marine nitrite-oxidizing bacteria (NOB) belong to three uncultured genera within the *Nitrospinaceae* family, and thus the ecophysiology of these main nitrate-producing microorganisms in the ocean remains poorly resolved. Here, we report on the environmental characterization of one of these genera, which we name *Candidatus* Nitromaricola. A single species within this genus, *Candidatus* Nitromaricola pontica, comprises >95% of the nitrite-oxidizing community in the permanently stratified Black Sea. This provided a unique opportunity to study this globally abundant genus *in situ,* across pronounced environmental gradients of oxygen and inorganic nitrogen concentrations. We show that *Ca*. Nitromaricola pontica is capable of oxidizing nitrite at similar rates in both oxic and apparently anoxic waters, although it had a higher abundance in the more oxygenated waters of the Black Sea. The genome of *Ca*. Nitromaricola pontica indicates that like other NOB, it is a chemolithoautotroph, oxidizing nitrite, and fixing inorganic carbon. However, *Ca*. Nitromaricola pontica and other members of the *Ca*. Nitromaricola genus lack canonical high affinity terminal oxidases found in some other NOB genera. Single-cell analyses confirmed active autotrophic growth and showed that ammonium and urea, rather than nitrite, were the main nitrogen sources for biomass production. Our findings identify *Ca*. Nitromaricola as a globally abundant marine NOB and highlight its cosmopolitan lifestyle.

## Introduction

In the oceans, nitrate is the most abundant form of fixed nitrogen (N), and its availability exerts a key control on marine primary production. The oxidation of nitrite to nitrate is largely mediated by aerobic nitrite-oxidizing bacteria (NOB). NOB are a polyphyletic group, with cultured representatives found in four phyla: *Pseudomonadota*, *Chloroflexota*, *Nitrospirota*, and *Nitrospinota* [1]. Marine NOB include the gammaproteobacterial genus *Nitrococcus* [2] and members of the phylum *Nitrospirota* [3, 4]. However, the vast majority of NOB in marine environments belong to the family *Nitrospinaceae* of the order *Nitrospinales*, within the phylum *Nitrospinota* [4–6]. There are four cultured representatives of the *Nitrospinaceae* family: *Nitrospina gracilis*, *Nitrospina watsonii*, *Candidatus* Nitrohelix vancouverensis, and *Ca*. Nitronauta litoralis, which were all isolated from marine environments [7–10]. Experimental and genomic analyses of these cultured *Nitrospinaceae* indicate that they are chemolithoautotrophs, which are dependent on nitrite oxidation for energy conservation via the enzyme nitrite oxidoreductase (NXR), and use electrons derived from this reaction to perform carbon fixation by the reductive tricarboxylic acid (rTCA) cycle [6, 7, 10]. All four cultured species are strict aerobes, and as such *N. gracilis* and *N. watsonii* encode a high affinity *cbb*_3_-type heme-copper cytochrome *c* oxidase (HCO). In contrast, *Ca*. Nitrohelix vancouverensis and *Ca*. Nitronauta litoralis lack canonical HCOs; thus, the identity of their terminal oxidase remains unclear [7, 10].

Available molecular data indicates that none of the four cultured representatives of the *Nitrospinaceae* family are abundant in the oceans. Instead, most marine NOB fall within three as-yet-uncultured genera within the *Nitrospinaceae*: *Ca*. Nitromaritima and two poorly characterized genera, which are sometimes referred to as “Nitrospinae Clade 2” [5, 11, 12]. Like the cultured *Nitrospinaceae*, the abundant uncultured marine *Nitrospinaceae* also appear to be aerobes; however, environmental reports have shown that some can thrive in low oxygen waters due to an extremely high affinity for oxygen [13, 14]. While other facets of their ecophysiology remain largely unexplored, metagenomic studies have indicated that many environmental *Nitrospinaceae* encode enzymes to assimilate urea (which are absent in the cultured representatives) and cyanate (only present in *N. gracilis*; [10, 11]). In the environment, Nitrospinae Clade 2 can potentially supplement up to half of their N-demand using these small dissolved organic compounds [15]. However, alternative energy generation pathways such as aerobic oxidation of H_2_ [16], heterotrophic growth [17], or sulfite oxidation [2, 18] that are present in other NOB have not been detected in the marine *Nitrospinaceae* [12, 15, 19].

Of the environmentally relevant marine *Nitrospinaceae*, the genus *Ca*. Nitromaritima has been frequently found in the low oxygen waters of the eastern tropical North and South Pacific Ocean, and the Arabian Sea [11, 12, 19], thus it has been suggested to be adapted to low oxygen environments [11, 12, 19]. The two Nitrospinae Clade 2 genera (which are represented by metagenome-assembled genomes currently assigned the placeholder names UBA8687 and LS-NOB in GTDB) occur in various marine environments [4–6]. They have been identified in the hypoxic coastal Gulf of Mexico [15], deep open ocean waters [5], marine sediments [20], brines [11], and sponges [21] and thus appear to be the main nitrite oxidizers in the oxic oceans. Yet despite the ubiquity of Nitrospinae Clade 2, little is known about their ecophysiology.

To investigate ecologically relevant marine nitrite oxidizers across gradients of oxygen and inorganic nitrogen, we conducted a research expedition to the Black Sea. The Black Sea is a permanently stratified marine basin with characteristic density layers [22, 23], where an ~50 m thick suboxic zone (where O_2_ concentrations are <10 μM) separates the well-oxygenated surface waters from a deep anoxic and sulfidic core [23]. Within this suboxic zone, ammonium concentrations are low, but oxidized nitrogen species, such as nitrite and nitrate, accumulate [23]. The predictable and well-constrained density layers within the Black Sea allow targeted sampling of water masses with different physico-chemical characteristics. Thus, the Black Sea provides an optimal environment to investigate nitrite oxidation and identify the key parameters which drive the activity and abundance of NOB in the environment.

## Materials and methods

### Sampling site

The western Basin of the Black Sea was sampled during cruise POS539 with the R/V Poseidon, from 6 November to 21 November 2019. Stable isotope incubations were carried out at six stations at three to four depths, and molecular and fluorescence *in situ* hybridization (FISH) analyses were done at two stations at four depths. The methods were described previously in detail [24, 25]. Incubation depths were defined by discrete density values and were chosen to represent the different redox conditions in the lower part of the oxycline, the nitrate maximum (~ σT 15.5 kg m^−3^), below the nitrate maximum (~ σT 15.8 kg m^−3^), and within ammonium-replete, but sulfide-deplete deep suboxic waters. Note, however, that in the deepest incubation depths at stations W1 and S3, sulfide was already detected at concentrations of ~4 and 8 μmol L^−1^, respectively.

Detailed information on sampling stations can be found in Table S1 [25].

### Process rate experiments

For nitrite oxidation rate experiments, seawater incubations were done for three to four depths per station. Incubations were set up headspace-free in 250 ml glass serum bottles and closed with degassed rubber stoppers as previously described [26, 27]. To minimize oxygen contamination, seawater was then bubbled with a gentle helium gas stream for 20 min. Subsequently, the water was amended with 5 μM ^15^N-nitrite, 5 μM ^14^N-nitrate, and 240 μM ^13^D-DIC. In one set of incubations, additional 10 μM oxygen were added and all incubations were aliquoted into 12 ml glass vials (Exetainers; Labco, UK). In all incubations, a glass vial each was sacrificed at five time points (after 0 h, 3 h, 6 h, 12 h, 24 h) by adding a glass bead and 100 μl of dilute HgCl_2_ (0.7 g/100 ml). After 24 h, 3 × 12 ml glass vials were pooled and the seawater fixed with 1% paraformaldehyde (12–24 h, 4°C) and filtered onto 0.22 μm gold-sputtered GTTP filters for nanoscale secondary-ion mass spectrometry (nanoSIMS) analysis (see below).

### ^15^N-rate measurements

Potential nitrite oxidation rates were determined from the increase in ^15^N-nitrate over time after addition of ^15^N-nitrite. For all time points, residual nitrite in 5 ml subsamples was removed with sulfamic acid; subsequently, spongy cadmium was used to reduce the nitrate to nitrite [28, 29], which was converted to N_2_O using acetic azide [30]. During this conversion, one N in the formed N_2_O is derived from the azide. Thus, we quantified the resultant ^45^N_2_O by gas chromatography isotope ratio mass spectrometry (GC-IRMS) with a customized TraceGas coupled to a multicollector IsoPrime100 (Isoprime, Manchester, UK). Linear regression slopes were calculated over all time points, and only slopes significantly different from zero are reported (*P*-value <0.05, one-sided Student *t-*test). The slopes were corrected for the initial labeling percentage of the added ^15^N-nitrite, which was determined by sulfamic acid conversion at the start of the incubation.

### DNA and RNA sampling, extraction, and sequencing

Samples for DNA and RNA analyses were collected at all depths of two stations (S3, W3) as described previously [24]. In brief, at each depth, around 4 L of water each was filtered onto two 0.22 μm cartridge filters (Sterivex, Millipore). The cartridge intended for DNA extraction was purged with air, while the one for RNA extraction was filled with RNAlater. Both were then frozen at −20°C until return to Bremen, where they were stored frozen at −80°C until extraction. DNA and RNA were extracted using DNeasy and RNeasy PowerWater kits, respectively (Qiagen, Germany), with an additional heating step at 65°C for 10 min before beat beating. For RNA extraction, we performed on-column DNase digestion. Metagenomes and metatranscriptomes were sequenced (paired-end mode, 2 × 150 bps) using MiSeq System (Illumina) at the Max Planck Sequencing Center in Cologne, Germany.

### Analysis of NOB community composition

Illumina adapter sequences were removed from quality-controlled paired-end reads with TrimGalore (v0.6.7, [31]) with Cutadapt (v4.0 with Python v.3.7.12, [32]). Subsequently, phyloFlash (v.3.4, SILVA database v138.1, [33, 34]) was run on the trimmed metagenomic reads to assess NOB abundance and community composition based on 16S rRNA gene reads in the metagenomes. Briefly, we used phyloFlash [33] to identify and reconstruct SSU rRNA sequences directly from the metagenomic reads. phyloFlash identifies SSU rRNA-containing reads by comparison against the SILVA database and subsequently assembles these reads into longer SSU rRNA sequences [33]. Sequences assigned to *Ca.* Nitromaricola were identified based on their phylogenetic affiliation within the LS-NOB genus, and their relative abundances were quantified using the total read counts assigned to this specific taxonomic lineage.

### Metagenome assembled genomes

Samples were assembled individually using MEGAHIT (v.1.2.9, [35]). Three samples (5158_A, 5158_D, 5158_H) were processed individually in SqueezeMeta (v.1.5.1) providing the MEGAHIT assembly (minimum contig length = 1000, [36]). SqueezeMeta’s automated binning pipeline produced two medium-quality (79.9% completeness, 7.9% contamination and 76.6% completeness, 4.3% contamination) and high-quality (96.5% completeness, 4.3% contamination; 96.5% completeness, 3.6% contamination according to CheckM v.1.2.2, [37]) metagenome assembled genomes (MAGs) assigned to NOB using CONCOCT (v.1.1.0, [38]). These two MAGs had an average nucleotide identity (ANI) >99.9% according to fastANI [39], and thus for the following analyses, only the lower contaminated one (contamination 3.6%) was used. The contigs of this MAG were iteratively (50 times) elongated by mapping the reads of one sample onto the contigs in the MAG with a minimum read identity of 95% and a minimum of aligned bases per read of 80%. More specifically, this was done by aligning the reads, belonging to the contigs within the incomplete MAG, using minimap2 (v.2.24-r1122, [40, 41]). Ultimately, the MAG was refined using anvi’o (v.7.1, [42]). The quality of our MAG and the published reference genomes was assessed with CheckM (v.1.2.2, [37]).

Genome annotations were done with prokka (v.1.14.6, [43]) and DRAM (v.1.5.0, [44]) that uses prodigal [45] for gene calling and KOfam [46], UniRef90 [47], Pfam [48], and dbCAN [49] for annotation.

Average amino acid identity (AAI) was calculated using CompareM (v0.1.2), and ANI was calculated using fastANI (v1.32, [39]).

### Identification of genes

Key genes were manually curated using DIAMOND (v.2.0.14.152, [50]) blastp searches of representative proteins and adding the genes to the respective reference alignments using mafft with the –add option (v7.487, [51]). For NxrA, a manually curated dataset as described below was used, and the NxrB database was constructed from all published *Nitrospinales* NxrB sequences on NCBI. To identify the *cbb*_3_-oxidase, the previously published heme-copper oxygen reductase (HCO) dataset was used to verify the DRAM annotation [52]. For identifying the cytochrome *bd* ubiquinol oxidase, the as-such-assigned sequences by DRAM [44] were added to the multiple sequence alignment (MSA2) of previously published CydA sequences [53]. Classification of the putative [NiFe] hydrogenases was done by extracting the sequence that was annotated by DRAM with the Pfam accession PF00374 and adding them to the database published as HydDB [54].

### NxrA database construction

To construct the NxrA database, genomes associated with *Nitrobacter*, *Nitrococcus*, *Nitrolancea*, *Nitrotoga*, *Nitrospirales*, and *Nitrospinales* were downloaded from NCBI as well as genomes published as *Candidatus* Nitrotheca, *Candidatus* Nitrocaldera [55], and *Candidatus* Nitrosedimenticola [56]. On obtained genomes, protein-coding genes were predicted using Prodigal (v2.6.3, [45]) and GTDB-Tk (v2.6.1, [57]) was used to assign taxonomic identity. NxrA sequences associated with *Nitrobacter*, *Nitrococcus*, *Nitrolancea*, *Nitrotoga*, *Nitrospira*, and *Nitrospina* were downloaded from NCBI and dereplicated at 99% identity using UCLUST (v11.0.667, [58]). Using DIAMOND (v2.0.14, [50]) blastp, the obtained NOB genomes were searched against the NxrA database, and only sequences with an identity >70% and a length >50 were kept. Seqkit (v2.12.0, [59]) was used to extract the matching part of the sequences, and the so obtained NxrA sequences were used in an hmmsearch (hmmer v3.1b2, http://hmmer.org; [60]) against the model PF00384.28, that encompasses Molybdopterin oxidoreductases. Only sequences with an e-value ≤10^−5^ and an expected accuracy per residue of the alignment >0.7 were kept. These sequences were used in an hmmscan (hmmer v3.1b2, http://hmmer.org; [60]) against the whole Pfam database [48], and sequences with the best hit to PF00384.28 and an e-value ≤10^−5^ were kept. NxrA sequences of *Candidatus* Nitrosediminicola were manually added to the dataset. NxrA is closely related to NarG, and therefore this NxrA dataset was used in a DIAMOND (v2.0.14, [50]) blastp search against a recently published NarG reference dataset [25]. NarG sequences that had a sequence identity >70% with the NxrA dataset were kept. The selected NarG sequences and the NxrA sequences together with *Pseudomonas* NarG as the outgroup were aligned using mafft (v7.526, [51]), trimmed using trimal (v1.5.rev1, option -automated1, [61]), and a phylogenetic tree was constructed using IQ-TREE (v3.0.1, -B 1000; -m MFP, [62]).

### Determination of NxrA read abundance

To quantify NxrA in our datasets, unassembled metagenomic reads were used in a DIAMOND (v2.0.14, [50]) blastx search against the NxrA database. Positive blastx matches were defined by an e-value <10^−6^, a bit score ≥50, an alignment length ≥30 amino acids, and a percent identity ≥40%. Reads were translated using seqkit (v2.12.0, [59]), and the correct translation was identified via a DIAMOND (v2.0.14, [50]) blastp search against the constructed dataset. Translated reads were added to the alignments of the constructed dataset using the --add-fragments option in mafft (v7.526, [51]) and placed into the tree using the evolutionary placement algorithm (EPA-ng v.0.3.8, [63]). The output was parsed using pplacer’s program guppy (v.1.1.alpha19-0-g807f6f3, [64]), and reads placed into the tree with a pendant length >0.1 were discarded. For each branch, the number of reads placed onto that branch was summed. The counts were normalized against the abundance of the housekeeping gene *rpoB*. This method confidently distinguishes NxrA reads at the order level, but not at the genus level.

### *Ca.* Nitromaricola pontica visualization by CARD-FISH

We used catalyzed reporter deposition fluorescence *in situ* hybridization (CARD-FISH) to visualize *Ca*. Nitromaricola pontica cells in our fixed seawater samples. The modified CARD-FISH protocol by Pernthaler and collegues [65] as published by Kitzinger and collegues [66] using horseradish peroxidase (HRP)-labeled probes was applied onto filtered and fixed seawater samples using oligonucleotide probes Ntspn759 [15] and Ntspn-Mod [5] at 15% formamide concentration together. Each CARD-FISH experiment included a positive control using probes EUB338 I-III [67, 68] and negative controls with the probe NonEUB [69] on separate filter pieces. Counting of target cells was done using an epifluorescence microscope (Axioplan 2, Zeiss, Germany).

### nanoSIMS determination of ^15^N-enrichment

We used samples from 104 m at station S3 analogous to Stuehrenberg *et al.* [24] and performed the established FISH-nanoSIMS protocol [66] with slight modifications. NanoSIMS filters were marked with the laser microdissection microscope (6000 B, Leica) prior to CARD-FISH hybridization. The nanoSIMS filter which was amended with ^15^N-ammonium was coated with an additional gold layer with a sputter coater (ACE600, Leica), prior to nanoSIMS measurement. The nanoSIMS instrument precision for detection of ^15^N/^14^N and ^13^C/^12^C isotope ratios was monitored regularly on Graphite Planchet. The samples were presputtered with a cesium (Cs^+^) primary ion beam of 300 pA at the nanoSIMS 50L (CAMECA). For measurement, the sample surface was rastered with a Cs^+^ primary ion beam (current: 1.5 pA) with a dwelling time of 1 ms per pixel for 40 planes (raster area 10 μm × 10 μm). The masses of 12C^−^, 13C^−^, 19F^−^, 12C14N^−^, 12C15N^−^, 31P^−^, and 32S^−^ were measured simultaneously, and the instrument was tuned for a mass resolving power >8000. The nominal spot size was <100 nm, and the image resolution was 256 px × 256 px. There were 23, 29, and 19 *Ca*. Nitromaricola cells from the Black Sea analyzed from incubations with ammonium, urea, and nitrite, respectively.

Subsequently, the nanoSIMS data was processed using the software Look@NanoSIMS (version 2025-02-13, [70]) in MATLAB (R2022b, The MathWorks Inc., 2022). The dead-time correction was applied and the registration-based algorithm was used for aligning nanoSIMS and fluorescence images. Only measurements with a Poisson error <5% across all planes were used for further calculations. The limit of detection for the ^15^N/(^14^N + ^15^N) enrichment was calculated for each filter (LOD = mean_background_ + 3 × sd_background_) and was 3.91 × 10^−3^, 3.79 × 10^−3^, 3.73 × 10^−3 15^N/(^14^N + ^15^N) on ammonium, urea, and nitrite, respectively. The cellular ^15^N-atom% excess was calculated as previously described [15]. Single-cell growth rates for significantly enriched single cells were calculated as previously described [71]. In brief for cells incubated with ^15^N-ammonium +^13^C-DIC, ^15^N-nitrite +^13^C-DIC, or ^15^N-urea +^13^C-DIC, respectively, single-cell growth rates were based on the incorporation of ^15^N into biomass assuming exponential growth and taking into account the labeling percentage of the added substrate (i.e. N-based growth rates). In the same incubations, C-based growth rates were calculated based on the incorporation of ^13^C (from the added ^13^C-DIC) into biomass assuming exponential growth and taking into account the labeling percentage of the DIC pool. In the ^15^N-ammonium incubations, C-based growth rates could not be determined because the ^13^C/^12^C ratio of the filter was consistently too high, likely caused by sample contamination, hindering a proper quantification of the ^13^C enrichment of the cells.

### Global abundance map of *Ca.* Nitromaricola

To assess the global abundance of *Ca.* Nitromaricola (previously provisionally named LS-NOB), the Sandpiper database ([72]; www.sandpiper.qut.edu.au) was searched for LS-NOB and filtered for marine or marine plankton metagenomes.

### Data analysis

Data was processed and visualized using MS Excel 2016 and RStudio (v4.4.3) including packages dplyr (v1.1.4), tidyr (v1.3.1), tidyverse (v2.0.0), patchwork (v1.3.0). All statistical tests were done in RStudio.

## Results

### Water column characteristics

We sampled the suboxic waters of the western basin of the Black Sea during a cruise on the R/V Poseidon (POS539) in November 2019 [24, 25]. Dissolved oxygen concentrations dropped below 10 μM at a density (σT) of 15.6–15.7 kg m^−3^ (Figs 1 and 2, Table S1), corresponding to water depths between 79 and 129 m, which is in accordance with previous observations [23, 73, 74]. The suboxic zone (defined as the layer where oxygen concentrations are <10 μM and sulfide concentrations are <1 μM) extended to a density of σT 16.21–16.3 kg m^−3^ (corresponding to 135–149 m water depth) at five out of six sampled stations (S2, S3, W1, W2, W3). At station P1, the lower boundary of the suboxic zone was not reached during sampling, and the oxygen concentrations at the deepest sampling depth were ~6 μM. Below the suboxic zone at the other stations, sulfide accumulation (concentrations >1 μM) was observed. As such the suboxic zone spanned over 30–56 m during our sampling campaign [25]. Broad nitrate concentration maxima ranging between 5.6 and 6.0 μM were observed at all stations and were located within the suboxic zone at four stations (P1, S2, S3, W3) and just above the suboxic zone at W1 and W2 (Figs 1 and 2, Table S1; [25]). Nitrate decreased to near zero in the lower part of the suboxic zone. This disappearance coincided with the appearance of ammonium at a density of σT 16.24–16.34 kg m^−3^ (ca. 135–149 m depth, Fig. 1), which then increased in concentration in deeper waters [24, 25]. Nitrite was detected at concentrations of up to 0.2 μM above the broad nitrate peaks (σT = 14.3–14.7 kg m^−3^) at four stations (W2, S2, S3, and P1), but otherwise nitrite concentrations were generally close to the limit of detection (Fig. 1, Supplementary Fig. 1, Table S1; [25]). Deep nitrite maxima, which have been observed sporadically within the suboxic zone at σT ~15.8 kg m^−3^ [23, 74], were not detected during this campaign. Urea concentrations exhibited no discernible trend with depth and ranged between 0 and 1.4 μM-N (median 130 nM-N, Fig. 1, Supplementary Fig. 2, Table S1; [24]).

**Figure 1:**
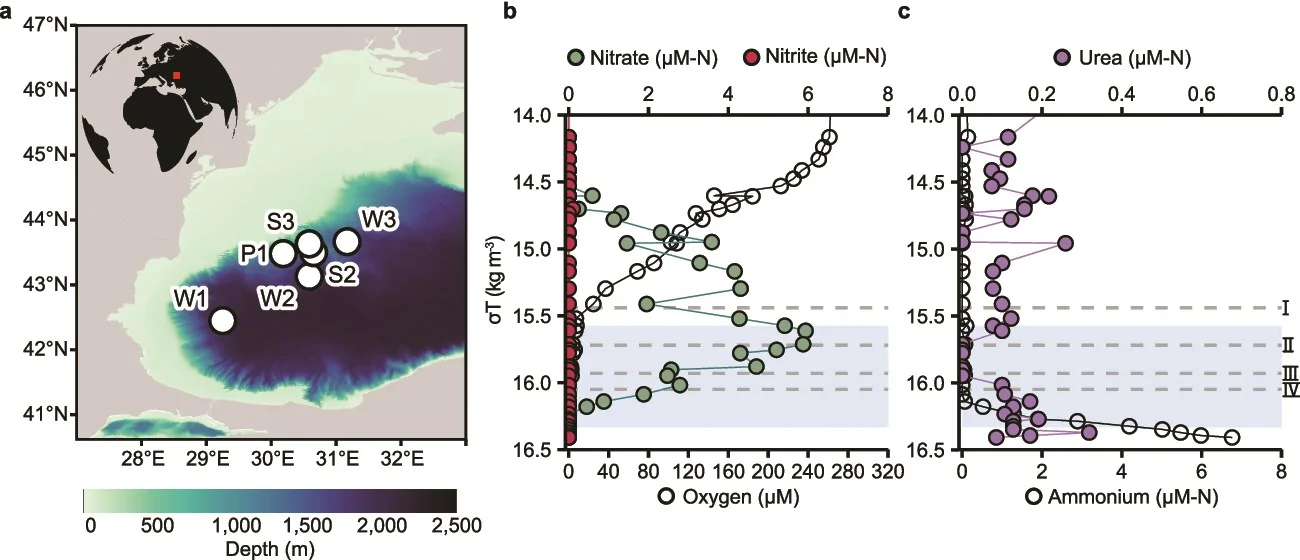
Study site and nutrient concentrations at a representative station in the western gyre of the Black Sea. (a) Map of the sampled stations depicting water depths (GEBCO 2024, doi: 10.5285/1c44ce99-0a0d-5f4f-e063-7086abc0ea0f). (b) *In situ* oxygen (open circles), nitrate (green), nitrite (red), (c) urea (purple), and ammonium (open circles) profiles at station W3. Shading indicates the suboxic zone, where the upper boundary is set by 10 μM O_2_, and the lower boundary is where sulfide concentrations increase above 1 μM. Dashed lines indicate incubation depths broadly coinciding with the lower part of the oxycline (I), the broad nitrate maxima (II), and with waters below the nitrate maxima, where ammonium had started to accumulate (III, IV). Nutrient profiles from all stations are published in von Arx *et al*. [25]; urea concentration profiles are shown in Supplementary Fig. 2.

**Figure 2:**
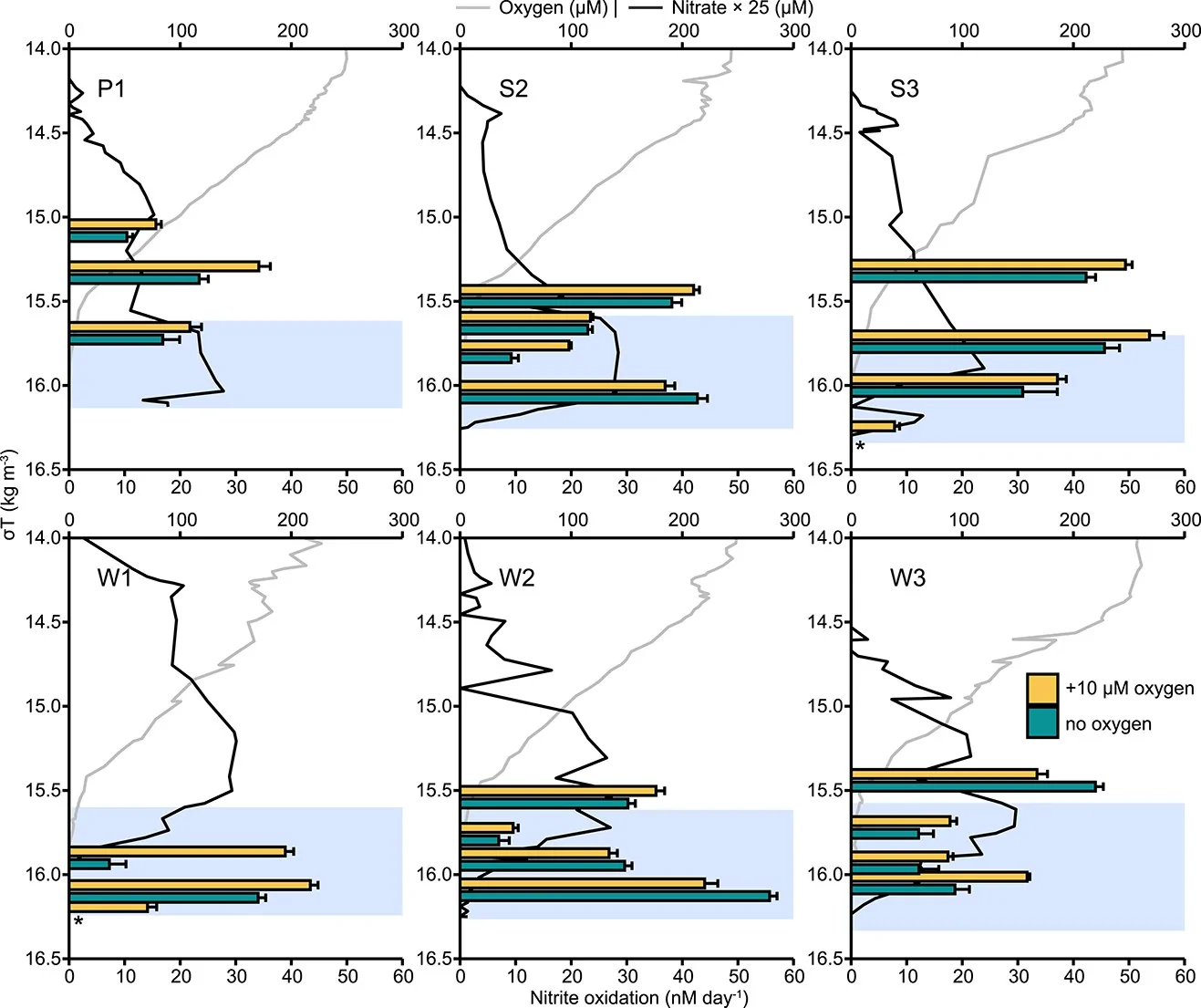
Potential nitrite oxidation rates in the suboxic zone of the western Black Sea. Each bar represents a statistically significant rate (one-sided Student’s *t*-test, *P* < .05) calculated from the slope across all time points, in incubations with 10 μM O_2_ (yellow) or in degassed incubations (green). Gray lines show oxygen; black lines show nitrate concentrations. Shading indicates the suboxic zone, where the upper boundary is set by 10 μM O_2_, and the lower boundary is where sulfide concentrations increase above 1 μM. Stars indicate incubation depths where sulfide was detected. Error bars indicate standard errors. At P1, no sulfide was detected, and the lower boundary represents the deepest depth sampled (σT = 16.13 kg m^−3^).

### Nitrite oxidation rates

Potential aerobic nitrite oxidation rates were assessed in incubations with the addition of 5 μM ^15^N-nitrite, 5 μM ^14^N-nitrate, and 10 μM oxygen (Supplementary Note). Incubations were performed at six stations in waters sampled from three to four depths, spanning from the lower part of the oxycline to the ammonium-containing waters at the base of the suboxic zone. At two stations (W1, S3) the lowest sampled depth contained sulfide (>1 μM). Linear rates of aerobic nitrite oxidation were quantifiable in all 22 incubations with no significant difference between stations. In the lower part of the oxycline (where O_2_ < 50 μM), rates ranged between 15.6 and 49.4 nM-N day^−1^. Within the broad nitrate maxima in the suboxic zone (O_2_ < 10 μM), they ranged between 17.8 and 53.7 nM-N day^−1^. Below the nitrate maxima, in waters where ammonium had started to accumulate, potential nitrite oxidation rates ranged between 9.5 and 43.4 nM-N day^−1^, and in the two depths at W1 and S3 where sulfide was present and O_2_ was <1 μM, rates were 14.1 and 7.8 nM-N day^−1^, respectively (Fig. 2, Supplementary Fig. 3, Table S2). Thus overall, there was no distinct pattern in potential nitrite oxidation rates associated with depth or density surface, with the exception of the sulfidic samples that had substantially lower rates than the other samples measured from the same stations (Fig. 2). The rates showed no correlation with nitrate or nitrite concentrations but were positively correlated with ammonia oxidation rates (published in Stuehrenberg *et al.* 2025 [24]) measured in parallel incubations (*R*^2^ = 0.58, *P*-value = 3.7 × 10^−5^, Supplementary Figs 4 and 5). However, potential nitrite oxidation rates consistently exceeded potential ammonia oxidation rates by ~2.8-fold (median).

As oxygen concentrations were often low (mostly <1 μM) in the deeper incubation depths, in a separate experiment, we tested whether nitrite oxidation was detectable in degassed incubations without oxygen addition. In 20 out of 22 incubations of these apparently anoxic incubations, linear rates of nitrite oxidation were quantifiable. In the lower part of the oxycline rates ranged from 10.4 to 55.7 nM-N day^−1^, in the broad nitrate maxima within the suboxic zone, they ranged between 9.2 and 45.6 nM-N day^−1^ (Fig. 2, Table S2). Below the nitrate maxima, potential nitrite oxidation rates ranged between 7.0 and 34.0 nM-N day^−1^, and in the two depths where sulfide was present, rates were not detectable (Fig. 2, Table S2). Overall, at the 20 depths with detectable rates, potential nitrite oxidation rates in the apparently anoxic incubations were not significantly different from the incubations that were supplemented with 10 μM oxygen (two-sided Student’s *t*-test, t = 1.2, df = 36.6, *P*-value = 0.26).

### Identity of nitrite-oxidizing bacteria in the Black Sea

To investigate the genomic identity of NOB in the Black Sea, we assembled draft MAGs from metagenome samples at station S3 and W3, the same depths where incubations were conducted. In total, four NOB MAGs were retrieved, of which two were medium quality (79.9% completeness, 7.9% contamination and 76.6% completeness, 4.3% contamination) and two were high quality (96.5% completeness, 4.3% contamination; 96.5% completeness, 3.6% contamination). The 79.9% complete medium-quality MAG belonged to the provisionally named genus UBA8687 within the Nitrospinae Clade 2 and had extremely low coverage, thus was not assessed further. The other medium-quality MAG, together with the two high-quality NOB Black Sea MAGs, shared an ANI of >98% and were thus dereplicated into one nearly complete MAG (96.5% completion with 2.6% redundancy, 3.0 Mb, GC 46%) that was used for further analysis.

The dereplicated Black Sea MAG clustered with 13 other publicly available genomes obtained from metagenome assemblies retrieved from various marine environments. Phylogenetic analysis revealed that this cluster, provisionally assigned as LS-NOB within GTDB, represents a distinct genus, with an average amino acid (AAI) of 57.7%–75.4% when compared to the other established *Nitrospinaceae* genera (*Nitrospina*, *Ca.* Nitromaritima, *Ca*. Nitrohelix, and *Ca*. Nitronauta), as well as the uncharacterized UBA8687 (Fig. 3, Table S3). Based on this, we now name this previously uncharacterized genus of marine NOB *Candidatus* Nitromaricola (see Discussion for etymology).

**Figure 3:**
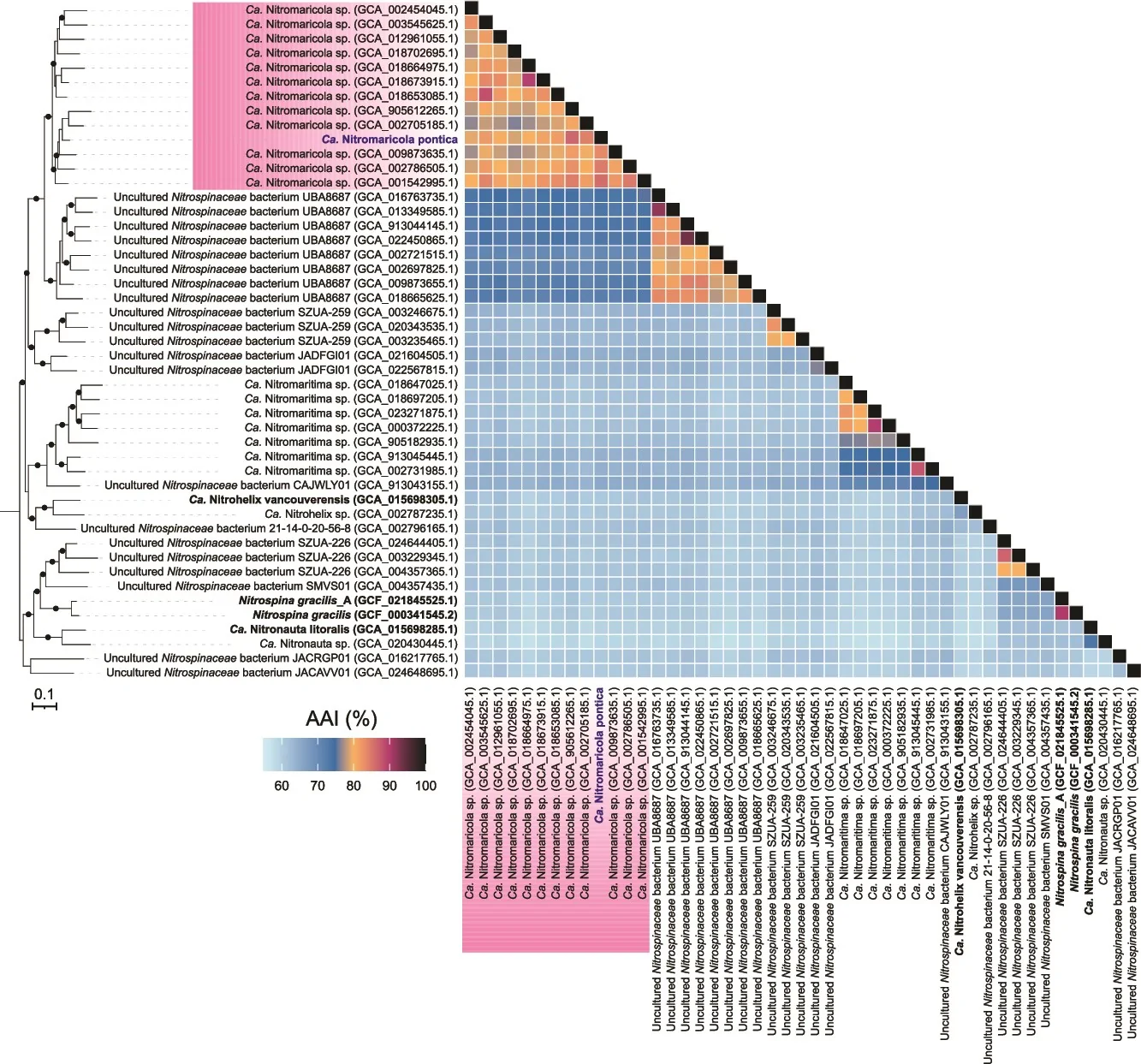
Maximum likelihood phylogenetic tree of concatenated bacterial marker genes in *Nitrospinales* (family *Nitrospinaceae*). Placement of MAG *Ca*. Nitromaricola pontica (bold, blue). Cultured representatives are bold and black. Genomes were dereplicated at 95%. Black dots indicate >95% bootstrap support. Scale bar indicates nucleotide substitutions per site. Phylogenetic tree was calculated using IQ-TREE2 [62], based on a PhyloPhlAn (v.3.1.68; [96]) alignment rerooted at *Nitrospirales* (not shown). Average AAI (≥55%) of *Nitrospinaceae* genomes are shown.

Pairwise genome comparison indicated that the Black Sea *Nitrospinaceae* MAG is closely related (ANI > 99%) to an uncharacterized high-quality MAG previously retrieved from suspended particulate matter in the Black Sea (GCA_014382285; [75]); thus, we consider them the same species (Supplementary Fig. 6). However, the Black Sea species is distinct from the remaining 12 genomes within the genus *Ca*. Nitromaricola, with ANI values ranging between 77.2%–83.0% (lower than the cut-off for species delineation, ANI > 95%; [76, 77]). In fact, all 12 of the other genomes within *Ca*. Nitromaricola are distinct species based on ANI values (Supplementary Fig. 6). Based on this, and our further environmental characterization (see below), we named the newly identified species from the Black Sea, *Candidatus* Nitromaricola pontica (see Discussion for etymology).

### Nitrite-oxidizing community composition and abundance in the Black Sea

Metagenomes were screened for the abundance of 16S rRNA genes associated with NOB at two of the six stations (S3, W3) in the same four depths where nitrite oxidation rates were determined. At both stations (S3, W3) and across all depths, >99% of the NOB belonged to the family *Nitrospinaceae*. Of these, 95% of the 16S rRNA reads were assigned to *Ca*. Nitromaricola; together with the metagenomic results, this shows that *Ca*. Nitromaricola pontica dominates the NOB community in the Black Sea (Fig. 4, Table S4). At both stations, the relative abundance of *Ca*. Nitromaricola pontica 16S rRNA reads was highest in the shallowest sampled depth (1.0% and 1.2% of total 16S rRNA reads, at S3 and W3, respectively), which was located within the lower part of the oxycline (Fig. 4). Within the nitrate maximum, the relative abundance of *Ca*. Nitromaricola pontica 16S rRNA reads was slightly lower (0.6% and 0.9%, at S3 and W3, respectively), decreasing further below the nitrate maximum (0.2% and 0.7%, at S3 and W3, respectively). In the deepest sampled waters at both stations, the relative abundance of *Ca*. Nitromaricola pontica 16S rRNA reads remained similar (0.3% and 0.7% at S3 and W3, respectively), even though at station S3, the deepest sample (131 m) was taken within sulfidic waters. Additionally, the abundance of NOB was investigated by quantifying reads assigned to NxrA, which encodes the alpha subunit of the NXR. The abundance and transcription patterns of NxrA were similar to that of the 16S rRNA reads (Supplementary Fig. 7, Tables S5–S7). Moreover, all metagenomic and metatranscriptomic NOB NxrA fragments were affiliated with the order *Nitrospinales*; however, further refinement of the taxonomic identity was not possible, due to the high identity of *Nitrospinales* NxrA sequences.

**Figure 4:**
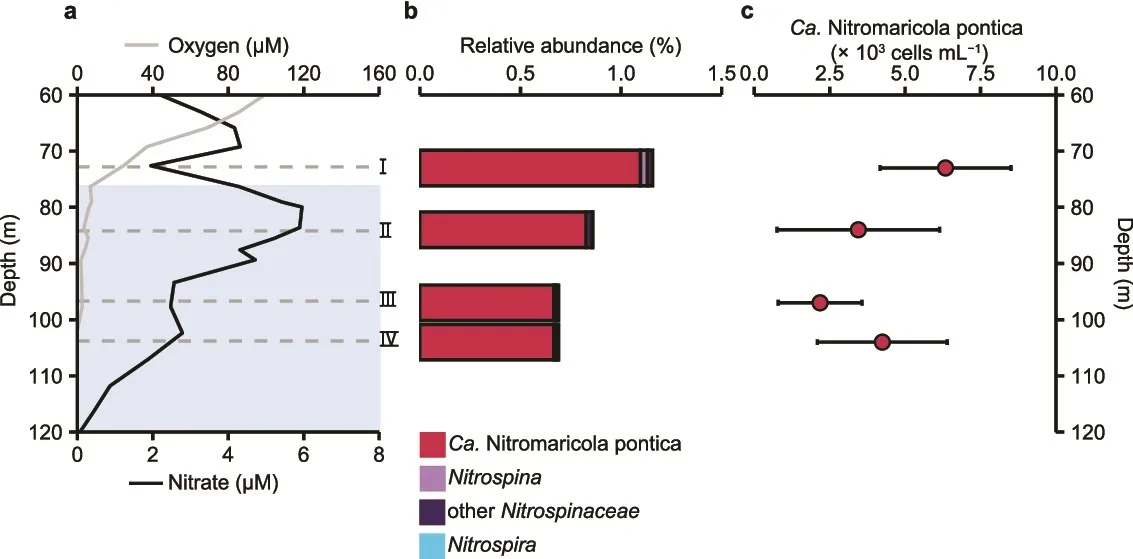
Community composition and abundance of NOB. (a) Oxygen (gray) and nitrate (black) concentrations in the Black Sea at station W3. Shading indicates the suboxic zone, where the upper boundary is set by 10 μM O_2_, and the lower boundary is where sulfide concentrations increase above 1 μM. Dashed lines indicate incubation depths broadly coinciding with the lower part of the oxycline (I), the broad nitrate maxima (II), and with waters below the nitrate maxima, where ammonium had started to accumulate (III, IV). (b) 16S rRNA gene abundance of different NOB within the *Nitrospinales* (red - *Ca*. Nitromaricola pontica, light purple - *Nitrospina*, dark purple - other *Nitrospinaceae*) and the *Nitrospirales* (blue - *Nitrospira*) in the Black Sea at station W3 based on phyloFlash. (c) *Ca*. Nitromaricola pontica CARD-FISH counts at station W3. Error bars indicate standard deviation of counts across different fields of view.

### Genetic capacity of *Ca*. Nitromaricola pontica and the genus *Ca*. Nitromaricola

We assessed the genetic capacity of *Ca*. Nitromaricola pontica as well as the other species within the genus, which have so far been largely uncharacterized. Of the 12 other species within the genus *Ca*. Nitromaricola*,* four were represented by high-quality MAGs (completion >90%, contamination <5%) and were thus included in the analysis. Two of these genomes were assembled from sponge metagenomes, whereas the remaining two were assembled from the seasonally hypoxic waters of the Gulf of Mexico and Saanich Inlet (Supplementary Fig. 6). In the Black Sea, *Ca*. Nitromaricola pontica encodes and transcribes both *nxrA* and *nxrB* but lacks *nxrC*, coding for the alpha, beta, and gamma subunit, respectively, of the NXR, the enzyme responsible for oxidation of nitrite to nitrate (Tables S8–S10; [78]). All of the four other genomes in the genus *Ca*. Nitromaricola encoded *nxrA*; however, only two other *Ca*. Nitromaricola genomes encoded *nxrB*, and none encoded *nxrC* (Fig. 5). Likely this is because the frequent presence of closely related gene variants in nitrite oxidizers makes accurate binning of *nxrABC* based on sequence coverage challenging [6].

**Figure 5:**
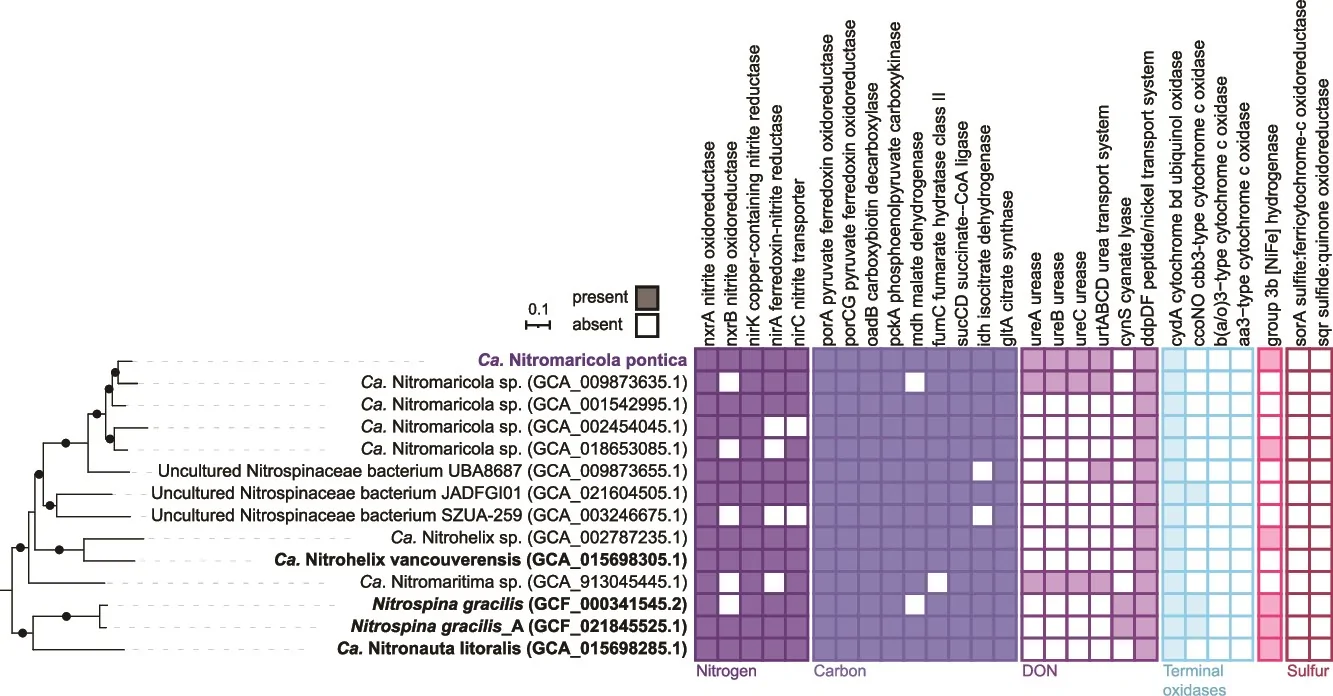
Presence/absence (filled/empty) map showing key genes of high-quality (completeness >90%, contamination <5%) *Nitrospinaceae* genomes. Phylogenomic tree based of high-quality genomes was calculated on an alignment by anvio’9 [42] using IQ-TREE2 [62]. Black dots indicate >95% bootstrap support. Cultured representative names are shown in bold.

The genome of *Ca*. Nitromaricola pontica encodes most of the genes (namely pyruvate ferredoxin oxidoreductase alpha/gamma subunit - *porACG*, carboxybiotin decarboxylase - *oadB*, phosphoenolpyruvate carboxykinase - *pckA*, malate dehydrogenase - *mdh*, fumarate hydratase, class II - *fumC*, succinate--CoA ligase [ADP-forming] subunit beta - *sucCD*, isocitrate dehydrogenase [NADP] - *idh*, citrate synthase - *gltA*) for CO_2_ fixation via the rTCA cycle (Fig. 5, Table S8), which is the common CO_2_ fixation pathway in *Nitrospinaceae* [7, 10]. The other four genomes also encoded these genes, with the exception of the Gulf of Mexico genome, which was missing *mdh* and *idh*. *Candidatus* Nitromaricola pontica also encodes and transcribes a copper containing nitrite reductase (*nirK*), as do the four other *Ca*. Nitromaricola species and all other sequenced nitrite oxidizers (e.g. [7, 10, 15, 19]; Fig. 5, Tables S8 and S10). The *Ca*. Nitromaricola pontica genome encodes an assimilatory ferredoxin-nitrite reductase (*nirA*) located next to a nitrite transporter (*nirC*), like *N. gracilis* [10]. *NirA* and *nirC* are also present in three of the four other *Ca*. Nitromaricola genomes. Additionally, *Ca*. Nitromaricola pontica encodes and transcribes genes (*ureABC*) that support the assimilation of urea-derived nitrogen, a feature that is only present in one other *Ca*. Nitromaricola species (from the Gulf of Mexico; Fig. 5, Tables S8 and S10). Although Ca. Nitromaricola pontica and the four other *Ca*. Nitromaricola species do not encode *cynS* (which is associated with cyanate utilization), they all encode additional genes that might enable uptake of organic N-compounds for growth, such as an amino acid and (oligo-)peptide ABC-type transporters (Table S8).

We further investigated whether *Ca*. Nitromaricola pontica has specific adaptations for the low oxygen and potentially sulfidic conditions that characterize the Black Sea. Its genome as well as the genomes of the other *Ca.* Nitromaricola species did not encode the high affinity *cbb*_3_ cytochrome *c* terminal oxidase found in *N. gracilis*. Nor did we find evidence for the presence of the high affinity b(a/o)3-type cytochrome *c* oxidase encoded by *Ca*. Nitromaritima MAGs from the eastern tropical South Pacific oxygen minimum zone [12] or the low-affinity *aa_3_*-type cytochrome *c* oxidase found in *Nitrococcus* genomes from the Namibian oxygen minimum zone [2]. However, the genome of *Ca*. Nitromaricola pontica, as well as those from the other *Ca*. Nitromaricola species, did encode subunit I of the so-called “*bd*-like oxidase” (Fig. 5, Tables S8–S10). This gene is highly conserved in all cultured *Nitrospinaceae* [7, 10, 79] and has previously been hypothesized to act as a terminal electron acceptor [7, 10]; however, its potential oxygen affinity is unknown.

Some NOB have previously been shown to have alternative energy generation pathways. *Nitrococcus*, *Nitrotoga*, and *N. gracilis* possess the genetic potential for sulfite oxidation [2, 10, 18], and some *Nitrospira* species have been shown to sustain chemolithoautotrophic growth by aerobic oxidation of H_2_ [16]. Generally, there was little genetic potential for sulfite oxidation within the genus *Ca*. Nitromaricola (i.e. no sulfite:cytochrome c oxidoreductase were identified). However, *Ca*. Nitromaricola pontica does encode and transcribe a group 3b [NiFe] hydrogenase, which is also present in the Saanich Inlet Ca. Nitromaricola genome (Fig. 5, Table S8), *N. gracilis*, and some other *Nitrospinaceae*, but is absent from *Ca*. Nitromaritima [12, 19, 80]. This bidirectional hydrogenase might enable aerobic hydrogen utilization or alternatively be involved in the reduction of polysulfides or elemental sulfur to hydrogen sulfide [6, 7, 10, 11, 80–82]. Overall, the genomic repertoire of the genus *Ca*. Nitromaricola is similar to that of other *Nitrospinaceae* genera.

### Visualization of *Ca*. Nitromaricola pontica in the Black Sea

To quantify the absolute cell abundances of *Ca*. Nitromaricola pontica *in situ*, we used CARD-FISH with the oligonucleotide probes Ntspn759 [15] and Ntspn-mod [5]. Broadly, these probes target the order *Nitrospinales,* but based on a blastn search of the respective probe against the small-subunit 16S rRNA genes within our samples, 93% and 97% (for Ntspn759 and Ntspn-mod, respectively) of the targeted genes were affiliated with *Ca*. Nitromaricola (Tables S11 and S12, Supplementary Fig. 8). Because the MAG data showed that *Ca*. Nitromaricola pontica is the only member of *Ca*. Nitromaricola within the Black Sea, this indicates that the probe was mainly targeting this species. The CARD-FISH cell counts were conducted at the two stations (S3, W3) where metagenomic and metatranscriptomic analyses were performed. Ca. Nitromaricola pontica had cell abundances of 6.3 × 10^3^ cells ml^−1^ to 6.7 × 10^3^ cells ml^−1^ at the shallowest depth within the lower oxycline, corresponding to 1.5% and 1.1% of the total microbial community at station W3 and S3, respectively (Fig. 4, Table S13). Within the nitrate maxima, *Ca*. Nitromaricola pontica cell numbers were lower than in the waters above (3.5 × 10^3^ cells ml^−1^ at W3 and 4.5 × 10^3^ cells ml^−1^ at S3). Below this depth, cell numbers further decreased at both stations. At W3, *Ca*. Nitromaricola pontica cell abundances were 2.2 × 10^3^ cells ml^−1^ below the nitrate maximum and 4.3 × 10^3^ cells ml^−1^ at the base of the suboxic zone. At S3, abundances were 4.5 × 10^3^ below the nitrate maximum and reached a minimum of 2.5 × 10^3^ cells ml^−1^ at the base of the suboxic zone, where sulfide was detected. Thus, the absolute counts of *Ca*. Nitromaricola pontica were slightly higher in the lower part of the oxycline, and decreased with depth, consistent with the patterns of 16S rRNA and *nxrA* gene read abundance in the metagenomes and metatranscriptomes. Because cell abundances decrease with depth, *Ca*. Nitromaricola pontica cell counts were positively correlated with *in situ* oxygen concentrations (Pearson correlation, t = 3.5, df = 6, *P*-value = 0.01; Supplementary Fig. 9).

### *In situ* N- and C-assimilation rates of *Ca*. Nitromaricola pontica

To verify the *in situ* activity of *Ca*. Nitromaricola pontica in the Black Sea and quantify their single-cell nitrogen and carbon assimilation, as well as calculate their growth rates, CARD-FISH-nanoSIMS was performed [66]. We quantified single-cell assimilation of ^15^N-ammonium, ^15^N-urea, and ^15^N-nitrite, as well as ^13^C-DIC by *Ca*. Nitromaricola pontica in O_2_ amended incubations carried out from the lower oxycline at station S3. In the incubations with ^15^N-ammonium, 22 out of 23 *Ca*. Nitromaricola pontica cells actively assimilated ^15^N at an average rate of 40.4 ± 17.6 amol-N cell^−1^ day^−1^ (average ± SD across all cells; Fig. 5, Table S14). This is equivalent to an ammonium-based growth rate of 0.06 ± 0.02 day^−1^ (Fig. 6). In incubations with ^15^N-urea, all 29 measured *Ca*. Nitromaricola pontica cells actively assimilated ^15^N-urea (20.0 ± 10.4 amol-N cell^−1^ day^−1^, average ± SD, Fig. 5, Table S14). This corresponded to a urea-based growth rate of 0.034 ± 0.01 day^−1^, around half of the ammonium-based growth rate. In incubations with ^15^N-nitrite, 14 out of 19 cells were enriched in ^15^N above the limit of detection, and average assimilation was 0.1 ± 0.1 amol-N cell^−1^ day^−1^ (average ± SD; Fig. 6, Table S14), <1% of either the ammonium or urea assimilation rates.

**Figure 6:**
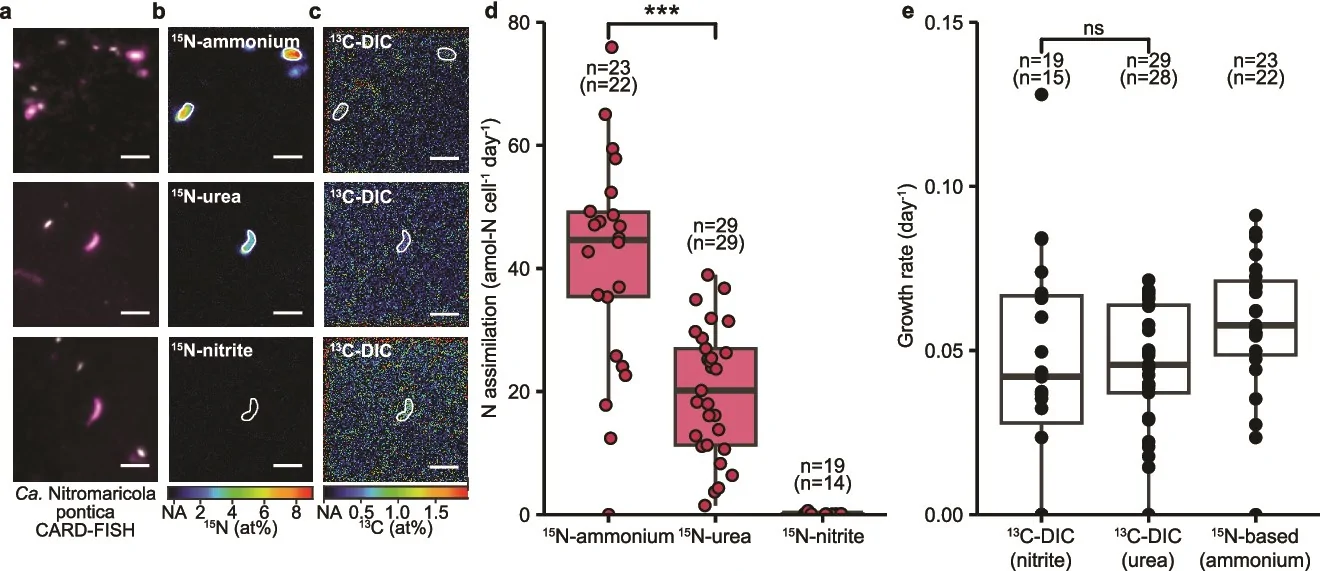
*Candidatus* Nitromaricola pontica single-cell activity. (a) Representative CARD-FISH images of *Ca*. Nitromaricola pontica (pink, stained by probe Ntspn759 + Ntspn_Mod) and other cells (white, stained by DAPI). (b) Corresponding nanoSIMS image of ^15^N at% enrichment after addition of ^15^N-ammonium, ^15^N-urea, or ^15^N-nitrite. (c) Corresponding nanoSIMS image of ^13^C at% enrichment after addition of ^13^C-DIC. *Candidatus* Nitromaricola pontica are marked by white outlines. Scale bar is 2 μm in all images. NA is the natural abundance and is 3.7 × 10^−3^ for ^15^N/(^14^N + ^15^N) and 1.06 × 10^−2^ for ^13^C/(^12^C + ^13^C). (d) N assimilation by *Ca*. Nitromaricola pontica cells from ^15^N-ammonium, ^15^N-urea, and ^15^N-nitrite. (e) Growth rates of *Ca*. Nitromaricola pontica cells based on ^13^C-bicarbonate assimilation in nitrite and urea incubations as well as based on ^15^N-ammonium. *n* = number of measurements per treatment with number of cells enriched significantly above the detection limit in brackets. ^***^/ns indicates significant/nonsignificant difference between treatments in two-sided Student’s *t*-test. N assimilation of ^15^N-ammonium was significantly higher than of ^15^N-urea (t = 4.9, df = 33.9, *P*-value = 2.34 × 10^−5^). The ^13^C-DIC growth rate was not different between incubations with ^15^N-nitrite and ^15^N-urea (t = −0.05, df = 25.4, *P*-value = 0.96).

Subsequently autotrophic growth rates of the *Ca*. Nitromaricola pontica were calculated based on the assimilation of ^13^C-DIC in the incubations containing ^15^N-nitrite and ^15^N-urea. In the ^15^N-ammonium incubations, C-based growth rates could not be determined (see Materials and methods), so growth rates were determined from ^15^N-ammonium assimilation. In the incubations carried out with ^15^N-nitrite and ^13^C-DIC, 15 out of 19 measured *Ca*. Nitromaricola cells were enriched in ^13^C-DIC at an average of 0.27 ± 0.19 at.% ^13^C excess (Supplementary Fig. 10, Table S14). This corresponds to a carbon-based growth rate of 0.046 ± 0.03 day^−1^ and a doubling time of ~22 days. In the incubations carried out with ^15^N-urea and ^13^C-DIC, 28 out of 29 *Ca*. Nitromaricola pontica cells were enriched at an average of 0.26 ± 0.10 at.% ^13^C excess, corresponding to a growth rate of 0.046 ± 0.02 day^−1^ and a doubling time of ~22 days. ^15^N-ammonium-based growth rates were 0.06 ± 0.02 day^−1^, corresponding to a doubling time of ~16 days. Overall, autotrophic (^13^C-DIC)-based growth rates in the ^15^N-nitrite and ^15^N-urea incubations were slightly, but significantly, lower (25%) than ^15^N-ammonium-based growth rates (two-sided Mann–Whitney–Wilcoxon tests; W = 371.5, *P*-value = 0.03, Fig. 6). There is the potential that ^13^C-enrichment measurements may be underestimated due to C-isotope dilution by the CARD-FISH procedure [83, 84].

## Discussion

### Persistent aerobic nitrite oxidation throughout the suboxic waters of the Black Sea

The permanently stratified Black Sea, characterized by well-defined gradients of oxygen and dissolved inorganic N, provides an optimal environment to investigate nitrite oxidation and the microorganisms involved at different oxygen and inorganic/organic N concentrations. Nitrite oxidation was detected throughout the suboxic zone of the western Black Sea, at potential rates ranging from 7.8 to 53.7 nM-N day^−1^ (Fig. 2), which are slightly lower than those reported previously from a different season (July) in a more central location in the Black Sea (between 50 and 240 nM-N day^−1^; [85]). Note that we refer to the rates as potential because the addition of ^15^N-NO_2_^−^ substantially increased nitrite availability above *in situ* concentrations and may therefore have stimulated nitrite oxidation relative to *in situ* rates (see Supplementary Note). An inhibitory effect of increased nitrite availability is considered unlikely as a previous study did not observe a decrease in nitrite oxidation rates at elevated substrate levels [14]. Overall, the potential nitrite oxidation rates reported in this study were consistently higher than corresponding potential ammonia oxidation rates (Supplementary Fig. 4), an offset in line with previous observations from the low oxygen waters of oceanic oxygen minimum zones [13, 85–91]. Because nitrite production by ammonia oxidation cannot fully support the nitrite demand of nitrite oxidation in the suboxic zone of the Black Sea, we suggest that this nitrite deficit is compensated for by nitrate reduction, which is supported by the ubiquitous presence of nitrate reduction genes at the study sites [25].

Generally, our potential nitrite oxidation rates from the Black Sea are comparable to those measured in the oxycline of the oxygen minimum zones of the eastern tropical North Pacific [14, 90, 92, 93] and eastern tropical South Pacific [88, 89, 91]. However, they are substantially lower than those measured in the low oxygen waters of the highly productive Gulf of Mexico [15] and coastal Benguela Upwelling System [29].

To mimic oxygen-limited conditions in the Black Sea suboxic zone, potential nitrite oxidation rates were also determined from degassed incubations. Generally, rates in the degassed incubations were comparable to those in the oxygen supplemented incubations (Fig. 2), except for two degassed incubations where no nitrite oxidation was detected. The absence of nitrite oxidation in these two degassed incubations coincided with depths where sulfide was present *in situ* (Fig. 2). The presence of sulfide likely either resulted in the consumption of the residual oxygen in the incubations, or inhibited the activity of the NOB and thus suppressed nitrite oxidation rates. In the remaining sulfide-free samples, nitrite oxidation was likely sustained by trace amounts of oxygen remaining in the experimental set-up, rather than an alternative electron acceptor. We base this on the stoichiometry of nitrite oxidation to oxygen consumption (2:1), which indicates that <30 nmol O_2_ L^−1^ would have been required to sustain the maximum nitrite oxidation rate measured in degassed incubations, as well as previous observations, which have shown that nitrite oxidation can occur at oxygen concentrations as low as 5 nM in oxygen minimum zones [13, 14]. These results imply that in the Black Sea, like in other low oxygen waters, nitrite oxidation can proceed under extremely low oxygen concentrations.

### Global abundance of the NOB genus *Ca*. Nitromaricola

We found that the Black Sea nitrite-oxidizing community was dominated by a single species, *Ca*. Nitromaricola pontica, which belongs to a previously uncharacterized genus within the *Nitrospinaceae* family. *Candidatus* Nitromaricola is phylogenetically distinct from *Ca*. Nitromaritima, the other uncultured, but partially characterized, marine *Nitrospinaceae* genus, which has been reported to dominate the low oxygen waters of the three major oxygen minimum zones [11, 12, 19] (Fig. 3).

To investigate the distribution and abundance of *Ca*. Nitromaricola outside of the Black Sea, we searched for members of this genus within a global metagenome database (Sandpiper; https://sandpiper.qut.edu.au/; [72]). Nitrite oxidizers of the *Ca*. Nitromaricola genus were detected in 2512 metagenomes annotated as marine or marine plankton. Across these metagenomes, the genus had a relative read-based abundance of 0.26 ± 0.32% (average ± SD) and a coverage of 8.8 ± 20.3 (average ± SD; Fig. 6, Table S15). In total, 1839 of the metagenomes carried geographical information, and the mapping of these datasets showed that the genus is globally distributed and can be found in both eutrophic coastal upwelling zones and oligotrophic open oceans, as well as across latitudes ranging from tropical to temperate to polar regions (Fig. 7). Relative abundances of up to 2.4% were identified in waters ranging in depth from the sunlit surface ocean to the bathypelagic zone (Table S15). This is in line with previous findings, which have shown that Nitrospinae Clade 2 (to which *Ca*. Nitromaricola belongs), together with *Ca*. Nitromaritima, are the most abundant NOB in pelagic marine environments [4, 5].

**Figure 7:**
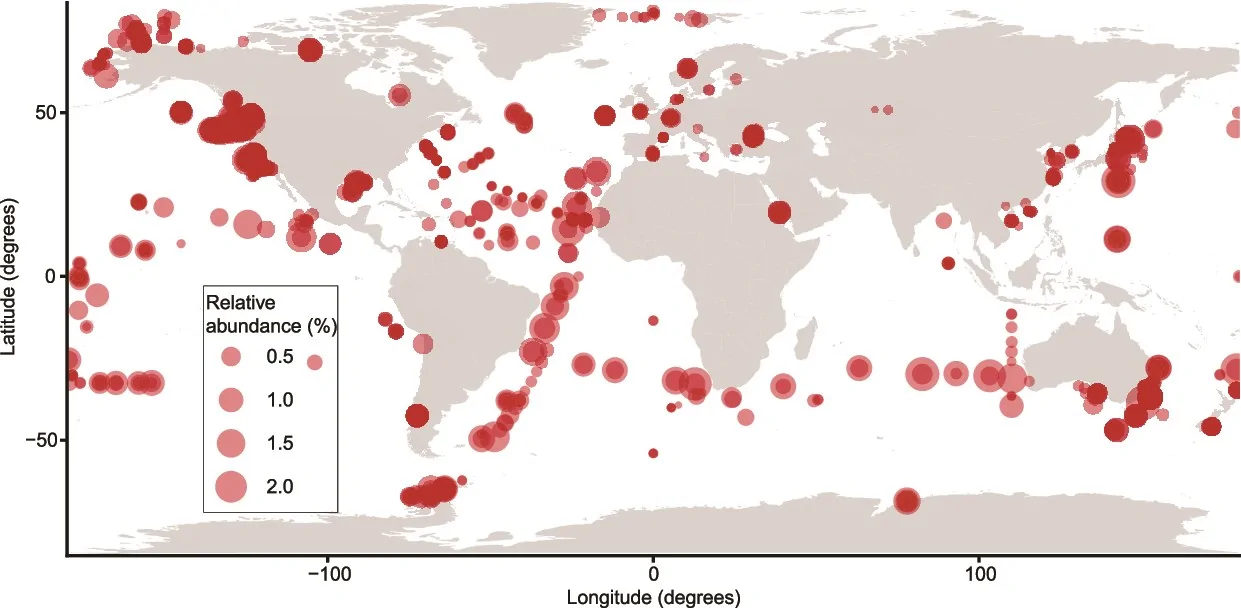
Global abundance of genus *Ca*. Nitromaricola (previously LS-NOB). Abundances and coverage of LS-NOB genus were retrieved from marine metagenomes using Sandpiper (https://sandpiper.qut.edu.au/; [72]). Circle size indicates relative abundance in respective metagenomes. Overlapping circles due to presence of genus in multiple datasets obtained from one location (or multiple locations in close vicinity) appear darker.

### Ecophysiology of the genus *Ca*. Nitromaricola

Members of the genus *Ca*. Nitromaricola remain uncultured; however, as the nitrite-oxidizing community in the Black Sea is composed almost exclusively of *Ca*. Nitromaricola pontica (95% based on 16S rRNA reads, Fig. 4), the region provided a model system that permitted characterization of the genus under *in situ* conditions. *Candidatus* Nitromaricola pontica was present throughout the investigated oxic to suboxic waters of the Black Sea, making up between 0.5% to 1.5% of the total microbial community (median 0.75%, Tables S4 and S13). These relative abundances are consistent with those of nitrite oxidizers reported from other marine habitats [5, 11, 12, 15, 29, 92]. Based on bulk and single-cell measurements as well as genome annotation*,* we show that *Ca*. Nitromaricola pontica oxidizes nitrite and fixes inorganic carbon using the enzyme NXR and the rTCA cycle, respectively, just like other *Nitrospinaceae* [7, 10].

*Candidatus* Nitromaricola pontica could assimilate ammonium, urea, and nitrite in the Black Sea (Fig. 6). However, their urea assimilation rates were around half of those observed for ammonium. Previous observations showed that a mixed Nitrospinae Clade 2 community, composed of *Ca*. Nitromaricola and UBA8687, assimilated urea to the same extent as ammonium [15]. Overall, these results show that organic-N can be an important growth substrate for members of the genus *Ca*. Nitromaricola. Compared to ammonium and urea, nitrite assimilation by *Ca*. Nitromaricola pontica is minimal (Fig. 6). The lack of detectable nitrite assimilation in incubations where nitrite was replete indicates that *Ca*. Nitromaricola pontica only uses nitrite as an energy source and not for growth, despite its genomic capacity for assimilatory nitrite reduction. This suggests that *Ca*. Nitromaricola pontica has regulatory mechanisms to control N-uptake pathways and competes successfully for reduced N-sources with other microorganisms, rather than use its primary energy source, nitrite, for growth.

Similar to other environmental NOB [13], *Ca*. Nitromaricola pontica appears to have a high affinity for oxygen, despite the absence of a high affinity *cbb*_3_-type heme-copper cytochrome *c* oxidase (or any other known terminal oxidase) in its genome. *Candidatus* Nitromaricola pontica and other members of the genus do, however, encode subunit I of the putative *bd*-like oxidase (Fig. 5). So far, this protein has not been biochemically characterized within the *Nitrospinaceae* but might function as a high affinity terminal oxidase [7, 10]. Despite the high affinity of *Ca*. Nitromaricola pontica for oxygen, we found a positive correlation between *Ca*. Nitromaricola pontica cell counts and *in situ* oxygen concentrations, indicating a slight preference for *Ca*. Nitromaricola pontica to occur in more oxygenated waters (Supplementary Fig. 9). Potentially, this preference is due to increased competition *in situ* for nitrite in the lower oxygen waters of the Black Sea, where microorganisms carrying out denitrification and anammox also co-occur [25, 73, 74, 94].

The per cell nitrite oxidation rate, and efficiency with which the energy gained from nitrite oxidation can be translated into carbon fixation, is a key determinant of how *Ca*. Nitromaricola pontica can succeed in the environment. On average, *Ca*. Nitromaricola pontica oxidized nitrite at a rate of 7.1 ± 2.4 fmol-N cell^−1^ day^−1^, which is slightly lower than single-cell oxidation rates reported for the mixed Nitrospinae Clade 2 community (including *Ca*. Nitromaricola) in the nutrient-rich Gulf of Mexico [15], but in line with per cell nitrite oxidation rates estimated from other *Nitrospinales* in the eastern tropical North Pacific [92]. Combined with the single-cell carbon fixation rates measured simultaneously (0.18 ± 0.1 fmol-C cell^−1^ day^−1^), we calculated that for every one mole of carbon fixed, *Ca*. Nitromaricola pontica need to oxidize ~39 moles of nitrite in the Black Sea, which is similar to cultured members of the genus *Nitrospina* [95].

### Ecological implications

NOB, belonging to three as-yet-uncultured genera within the *Nitrospinaceae* family, play a key role in the world’s oceans by producing nitrate. Although *Ca*. Nitromaritima is comparably well studied in oxygen minimum zones, very little is known about either of the two other genera that have been collectively referred to as Nitrospinae Clade 2. We characterized one of these poorly understood genera in the Black Sea, naming it *Ca*. Nitromaricola. The nitrite-oxidizing community was almost exclusively (>95%) composed of a of a single species, *Ca*. Nitromaricola pontica. This allowed us to study the ecophysiology of this globally abundant genus *in situ* under the varying oxygen and inorganic nitrogen conditions that are characteristic for the Black Sea. Intriguingly, neither the *in situ* observations, nor the analysis of *Ca*. Nitromaricola genomes revealed any specific adaptations that might drive niche differentiation between *Ca*. Nitromaricola and *Ca*. Nitromaritima, the genus often occurring in oxygen minimum zones. In fact, the ability of *Ca*. Nitromaricola to perform nitrite oxidation even at low-nanomolar oxygen concentrations and its presence in both the Black Sea and many oxygen minimum zones suggest that its range overlaps with that of *Ca*. Nitromaritima and also *Nitrococcus*, which has periodically been found in low oxygen marine waters. This suggests that oxygen alone does not determine the niche of marine NOB. Instead, the ability of *Ca*. Nitromaricola to also thrive in the shallower, more oxygenated waters of the Black Sea, as well as utilize urea as an additional nitrogen source, suggests that they are well adapted to a broad range of conditions. This adaptability might drive their cosmopolitan distribution in the oceans.

### Etymology

*Nitromaricola* L. n. *nitrum*: nitrate; L. neut. n. *mare*, maris the sea; *-cola*, dweller, from L. masc./fem. n. incola, an inhabitant, dweller; N.L. fem. n. *Nitromaricola* a nitrate inhabitant of the sea. pon’ti.ca. L. fem. adj. *pontica*, related to the Black Sea (note this is not N.L. as the term was used in antiquity, e.g. Ovid’s Epistulae ex Ponto).

## Supplementary Material

Supplementary_materials_wrag271

## Data Availability

All sequence data and the genome of *Ca*. Nitromaricola pontica generated in this study are deposited in NCBI under BioProject number: PRJNA1091553.
