# Supplementary material for: Ecophysiology of the cosmopolitan nitrite-oxidizing genus Candidatus Nitromaricola
Source: ISME J. 2026 Oct 19;20(1):wrag271. doi: 10.1093/ismejo/wrag271 (PMC13768755; doi:10.1093/ismejo/wrag271)
Supplement: Supplementary file 1 [file supplementary_materials_wrag271.zip › 261016_NOB_Black_Sea_supplementary_info.pdf]

Supplementary Information

Ecophysiology of the cosmopolitan nitrite-oxidising genus *Candidatus* Nitromaricola

Joerdis Stuehrenberg[1], Jan N. von Arx[1], Katharina Kitzinger[1,+], Gaute Lavik[1], Sten Littmann[1], Hannah K. Marchant[1,2,*], Jana Milucka[1], Luis H. Orellana[1], Marcel M. M. Kuypers[1]

1 Max Planck Institute for Marine Microbiology, Celsiusstrasse 1, 28359 Bremen, Germany

2 MARUM - Centre for Marine Environmental Sciences University of Bremen, Leobener Strasse 8, 28359 Bremen, Germany

+ Current address: Division of Microbial Ecology, Centre for Microbiology and Environmental Systems Science, University of Vienna, Djerassipl. 1, 1030 Vienna, Austria

*Corresponding author: hmarchan@mpi-bremen.de

Contents

Supplementary Note
Supplementary Figures
Supplementary Tables
Supplementary References

Supplementary Note

To quantify nitrite oxidation rates 5 µM ^{15}N-nitrite was added as tracer. Such nitrite additions were relatively large in comparison to the background in this system (up to 0.2 µM), and thus rates should be considered as potential, as they may have been stimulated by the addition. However, we consider that any substantial stimulation is unlikely for a number of reasons. First; the accumulation of ^{15}N-nitrate was linear over the course of the incubations, with no indication of an initial increase or progressive acceleration in rates following tracer addition. Second; our rates are lower than those determined previously in the Black Sea where only 200 nM ^{15}N-nitrite was added (Ward and Kilpatrick, 1991). Third; our rates are also comparable to those measured in other systems, where tracer additions were also much lower [1, 2].

Like the nitrite oxidation rates, the ammonia oxidation rates to which they are compared should also be considered potential rates, as they were determined based on 5 µM ^{15}N-ammonium addition, and ammonium concentrations were generally <0.2 µM in situ at the depths sampled for incubations. However, as the absolute tracer additions and tracer ratios were comparable for both ammonia and nitrite oxidation, we consider it unlikely that differential substrate stimulation explains the consistently higher nitrite oxidation rates, particularly given that this observation is consistent with observations from previous studies [1-8].

Supplementary Figures

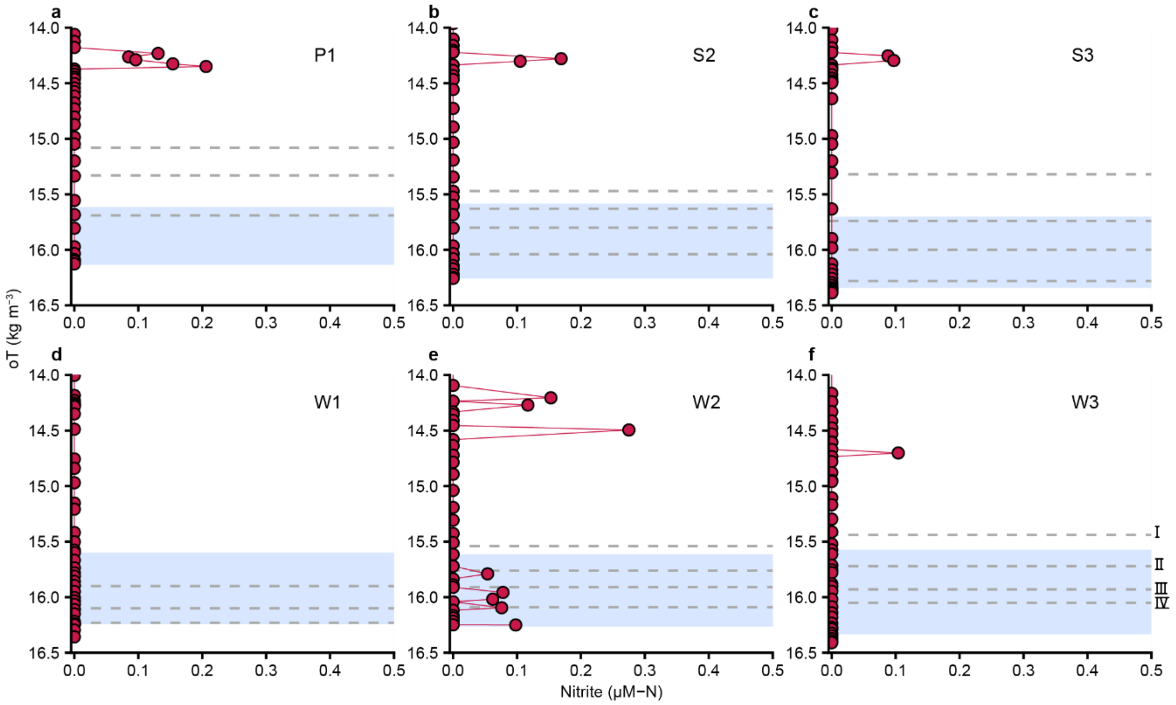

Supplementary Figure 1: Nitrite concentrations of station P1 (a), S2 (b), S3 (c), W1 (d), W2 (e) and W3 (f) in the western Black Sea. Grey dashed lines indicate incubation depths. Blue shading indicates the suboxic zone, where the upper boundary is 10 µM O_2, and the lower boundary is where sulfide concentrations increase above 1 µM. Grey dashed lines indicate incubation depths broadly coinciding with the lower part of the oxycline (I), the broad nitrate maxima (II) and with waters below the nitrate maxima, where ammonium had started to accumulate (III, IV).

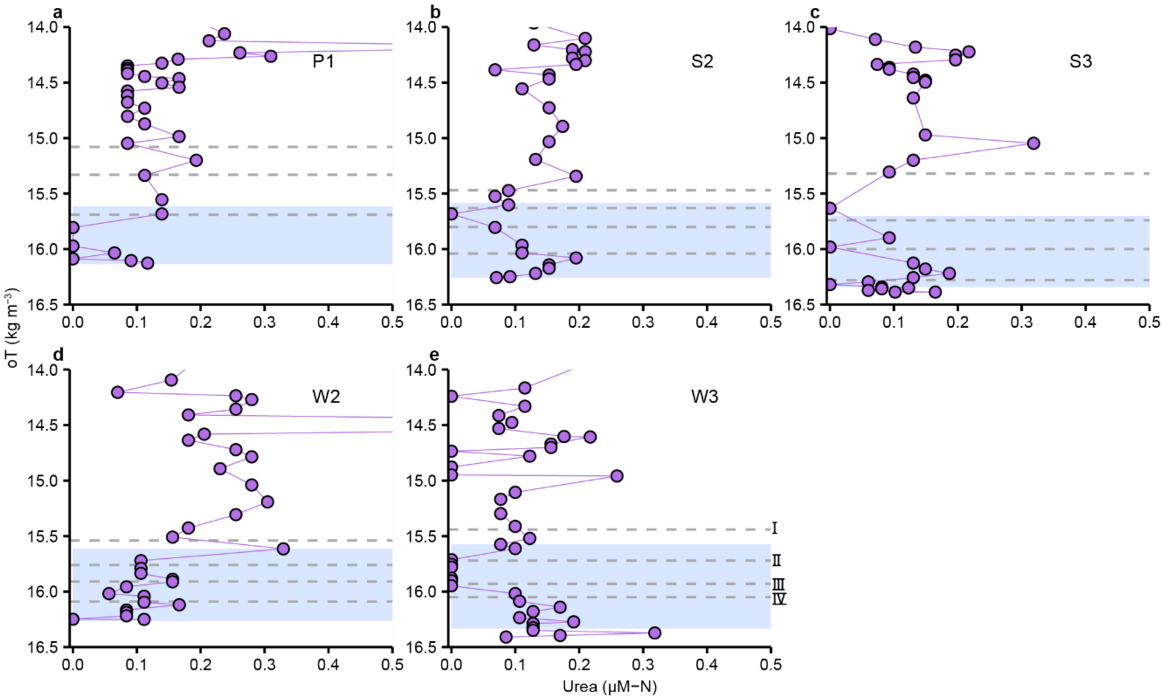

Supplementary Figure 2: Urea concentrations of station P1 (a), S2 (b), S3 (c), W2 (d) and W3 (e) in the western Black Sea. Grey dashed lines indicate incubation depths. Two urea concentrations are not depicted: 0.77 µM-N (station P1, 14.18 kg m^{-3}), 1.42 µM-N (station W2, 14.49 kg m^{-3}). Blue shading indicates the suboxic zone, where the upper boundary is 10 µM O_2, and the lower boundary is where sulfide concentrations increase above 1 µM. Grey dashed lines indicate incubation depths broadly coinciding with the lower part of the oxycline (I), the broad nitrate maxima (II) and with waters below the nitrate maxima, where ammonium had started to accumulate (III, IV). Measurements for station W1 are not available. Nutrient data is available in Table S1.

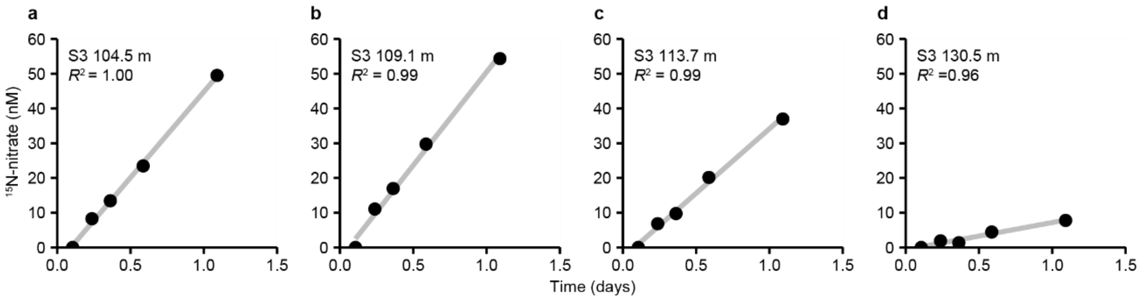

Supplementary Figure 3: Linearity of nitrite oxidation rates. Exemplary ^{15}N-nitrate concentration over time after addition of ^{15}N-nitrite and 10 µM O_2 in the Black Sea at station S3 104.5 m (a), 109.1 m (b), 113.7 m (c) and 130.5 m (d). Individual biological replicates were sacrificed per time point. Lines are linear regressions across all time points.

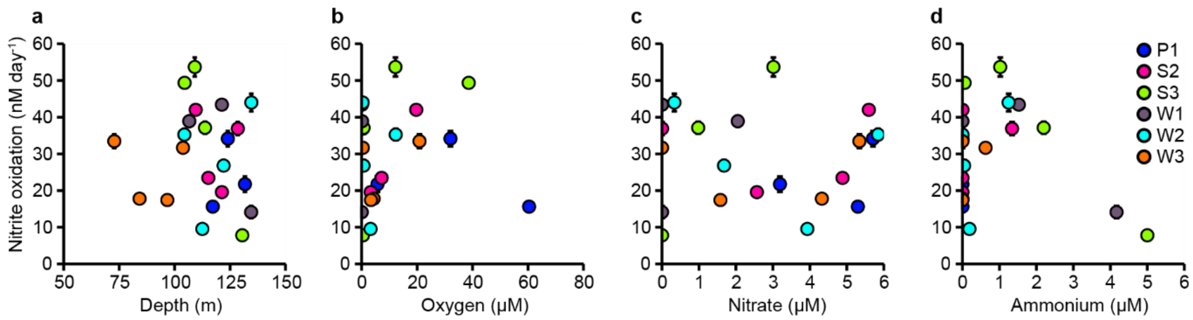

Supplementary Figure 4: Correlation of nitrite oxidation with different parameters. Correlation of nitrite oxidation rates over depth (a), or with *in situ* oxygen (b), nitrate (c), ammonium (d) concentration at station P1 (dark blue), S2 (pink), S3 (green), W1 (grey), W2 (light blue), W3 (orange).

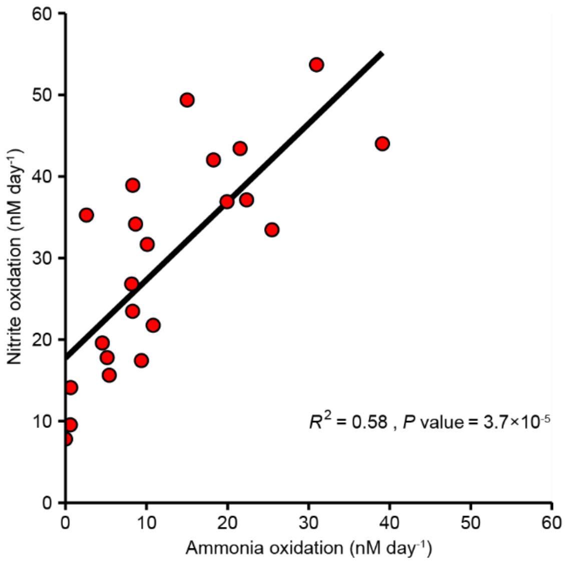

Supplementary Figure 5: Nitrite oxidation rates as a factor of ammonia oxidation rates in oxygen supplemented incubations. Each point represents two individual statistically significant rates calculated from the slope of all time points within the incubation (one-sided student's t-test, $p < 0,05$). One insignificant rate in the ammonia oxidation dataset (from within the sulfidic zone at S3) is plotted as zero.

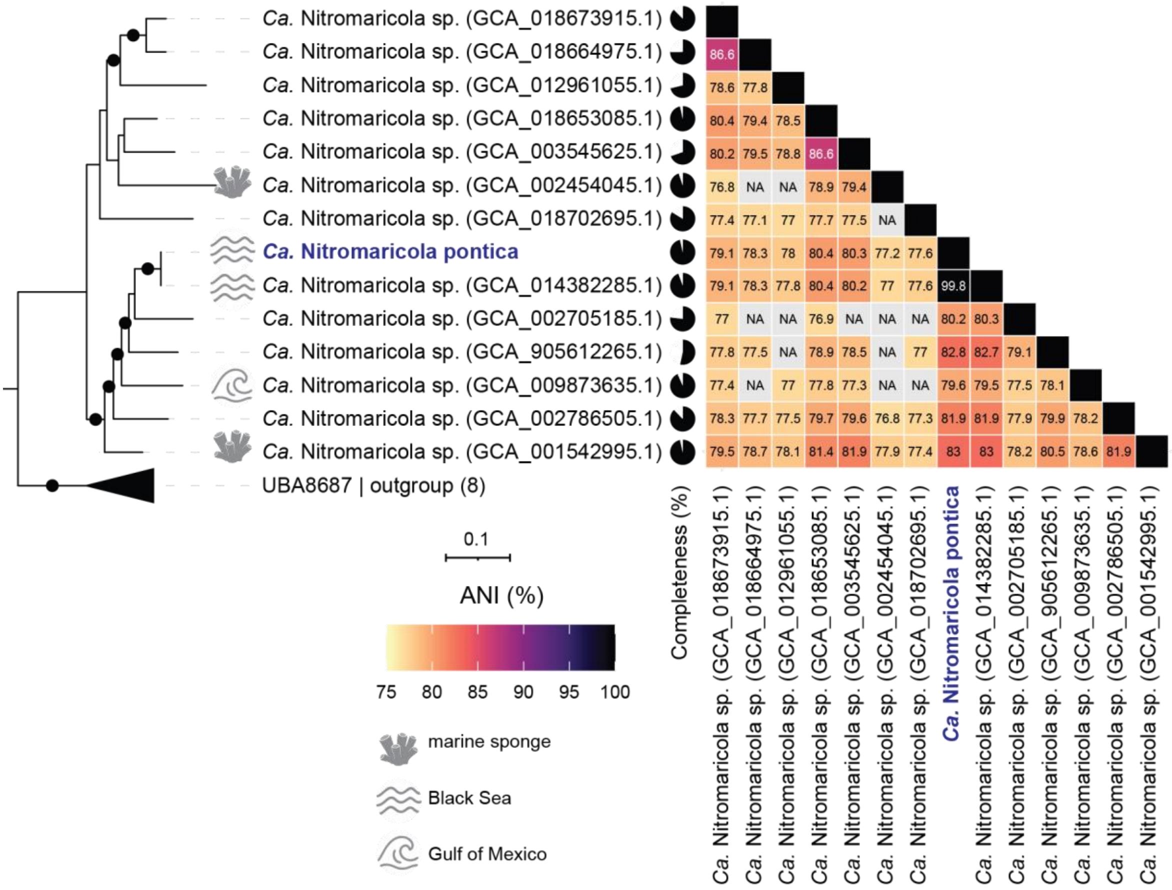

Supplementary Figure 6: Maximum likelihood phylogenetic tree of concatenated bacterial marker genes in *Ca.* Nitromaricola. The placement of *Ca.* Nitromaricola pontica is indicated in blue. Black dots indicate >95% bootstrap support. Scale bar indicates nucleotide substitutions per site. Phylogenetic tree was calculated using IQ-TREE2 [9], based on an anvio'9 alignment re-rooted at UBA8687. Average nucleotide identity (ANI ≥75%) of *Ca.* Nitromaricola genomes are shown. Completeness was assessed using CheckM (v1.2.2) and is depicted as pie charts. The environmental origin of the four high quality (completion >90%, contamination <5%) *Ca.* Nitromaricola genomes is indicated by icons.

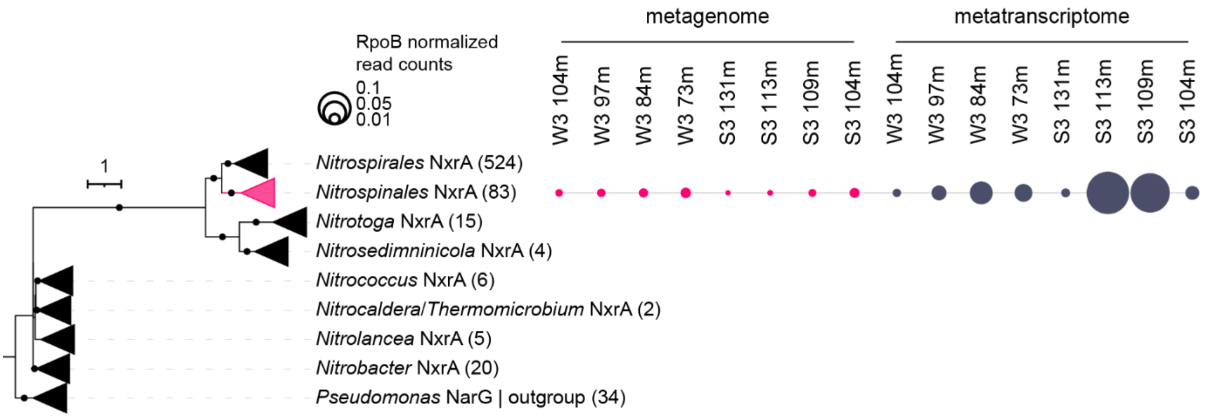

Supplementary Figure 7: NOB-affiliated NxrA and assembled amino acid sequences retrieved from the Black Sea (indicated by pink branch). RpoB normalized read counts (depicted as circles) were obtained by mapping metagenomic (pink) and metatranscriptomic (dark grey) reads from different stations (W3, S3) and depths from the Black Sea onto the alignment of target protein. Scale bar represents amino acid substitutions per site. Phylogenetic tree was calculated using IQ-TREE2 [9], model Q.pfam+R5, based on a mafft [10] alignment, trimmed using trimal [11] and re-rooted at *Pseudomonas* NarG. The abundance of Nitrospinales NxrA in metagenomic samples was generally higher in the lower oxycline and nitrate maxima (RpoB normalized read counts of 0.01, 0.008 at W3 and 0.009, 0.006 at S3, respectively) and decreased beneath the nitrate maxima and at the base of the suboxic zone (RpoB normalized read counts of 0.005, 0.002 for S3 and W3, respectively). The transcription of Nitrospinales NxrA followed a different pattern, increasing from RpoB normalized read counts of 0.03 and 0.02 (W3 and S3, respectively) in the lower oxycline to 0.05 and 0.18 in the nitrate maxima (W3 and S3, respectively). Below the nitrate maxima the transcription of Nitrospinales NxrA and - similar to the metagenomic abundance - was lowest in the deepest depth (RpoB normalized read counts of 0.006 and 0.007, W3 and S3, respectively). Data displayed here can be found in Table S7.

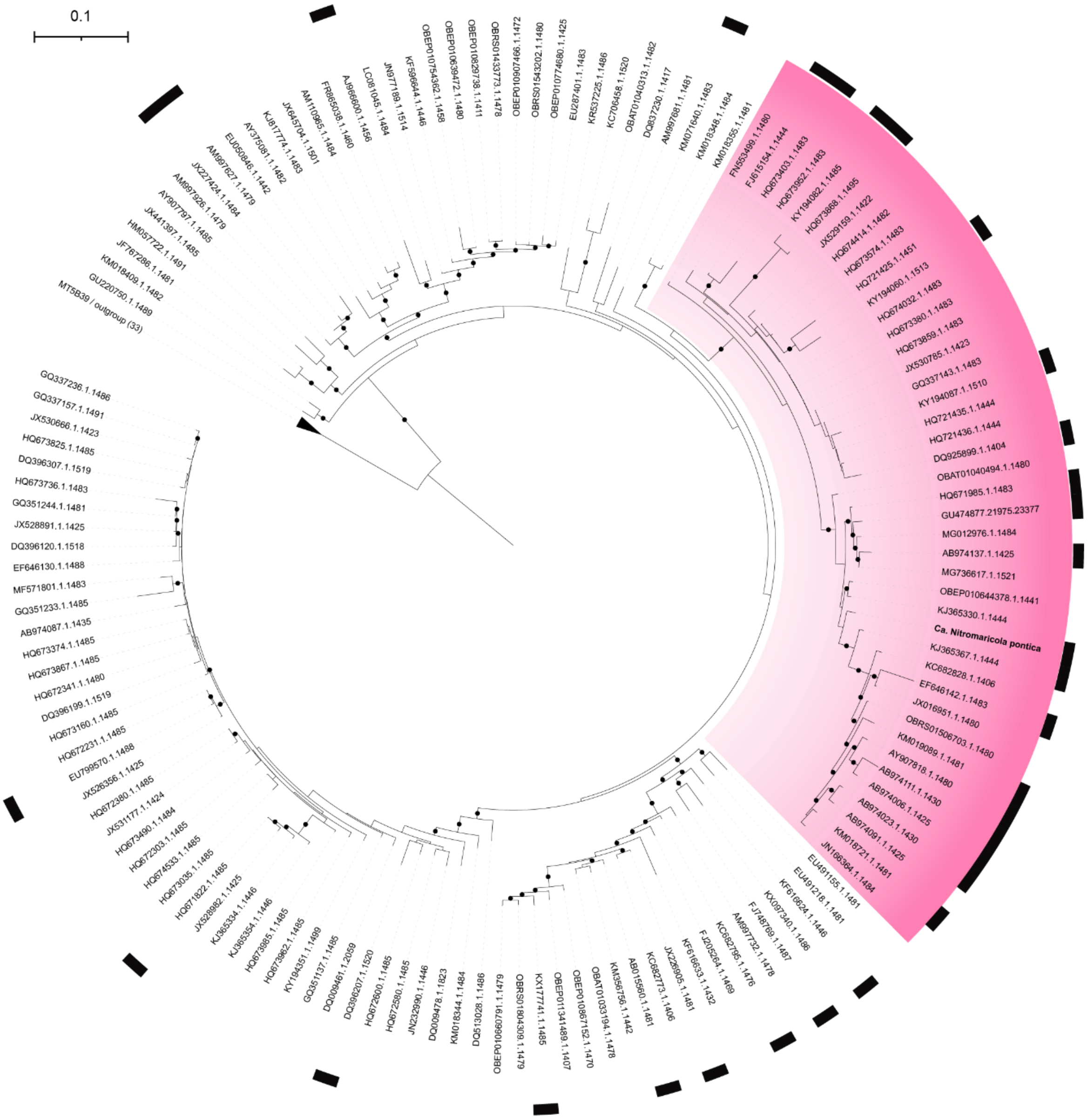

Supplementary Figure 8: 16S rRNA tree of the *Nitrospinia* and highlighted in pink is the genus *Ca.* Nitromaricola incl. the 16S rRNA sequence from our MAG in bold. Black rectangles show the sequences that have a perfect match with either CARD-FISH probe Ntspn759 or CARD-FISH probe Ntspn_mod. Scale bar represents nucleotide substitutions per site. Phylogenetic tree was calculated using IQ-TREE2 [9], model TIM2e+R4, based on a mafft [10] alignment, trimmed using trimal [11] and re-rooted at MT5B39.

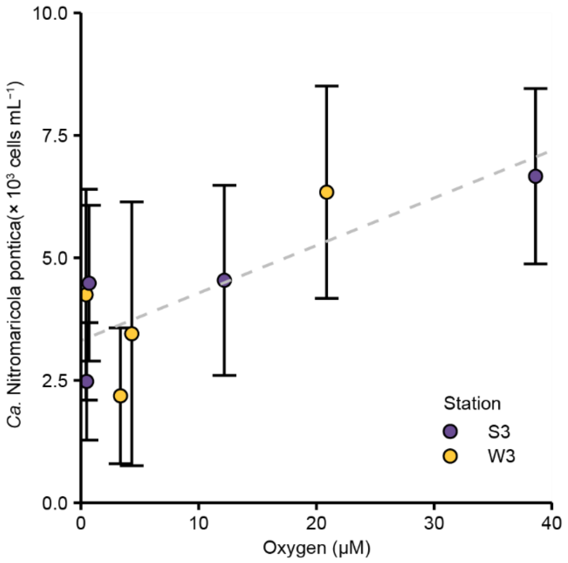

Supplementary Figure 9: *Ca.* Nitromaricola pontica cell counts against *in situ* oxygen concentrations at station S3 (purple) and W3 (yellow). Error bars represent the standard error. *Ca.* Nitromaricola pontica cell counts and *in situ* oxygen concentrations correlate significantly (grey dashed line, linear regression, multiple R^2 = 0.67, P value = 0.014, slope = 0.097, intercept = 3.3).

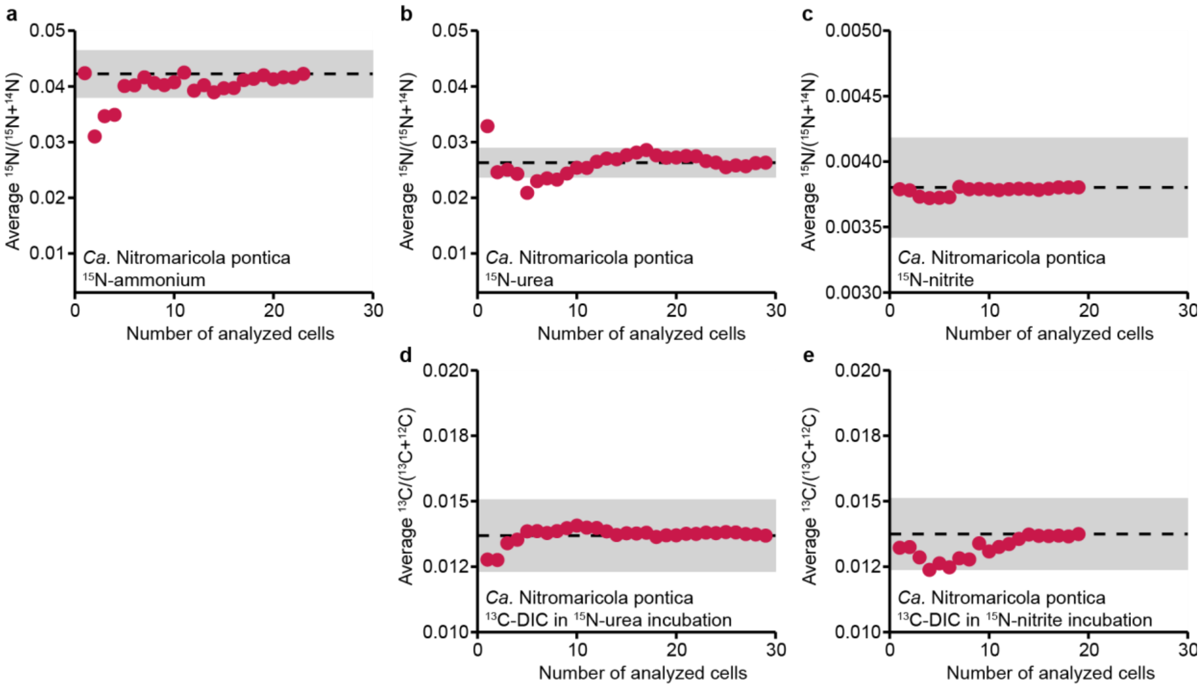

Supplementary Figure 10: Enrichment statistics of *Ca.* Nitromaricola pontica cells analyzed by NanoSIMS. Dots represent the means of $^{15}N/(^{14}N+^{15}N)$ ratios calculated across randomly subsampled cells. The black dashed line represents the mean across all cells, the light gray area ± 10% of the mean. Note that the scale for ^{15}N-nitrite is different. The total number of analyzed cells was a) n = 23, b) n = 29, c) n = 19, d) n = 29, e) n = 19. Note that the y-axis scale of panel c is different to the one in panel a and b.

Supplementary References

1. Peng X, Fuchsman CA, Jayakumar A *et al.* Ammonia and nitrite oxidation in the eastern tropical north pacific. *Global Biogeochem. Cycles*. 2015;**29**:2034-2049 https://doi.org/10.1002/2015gb005278
2. Santoro AE, Buchwald C, Knapp AN *et al.* Nitrification and nitrous oxide production in the offshore waters of the eastern tropical south pacific. *Global Biogeochem. Cycles*. 2021;**35** https://doi.org/10.1029/2020gb006716
3. Ward BB, Kilpatrick KA Nitrogen transformations in the oxic layer of permanent anoxic basins: The black sea and the cariaco trench. *Springer.* 1991;**351** https://doi.org/10.1007/978-94-011-2608-3_7
4. Bristow LA, Dalsgaard T, Tiano L *et al.* Ammonium and nitrite oxidation at nanomolar oxygen concentrations in oxygen minimum zone waters. *Proc. Natl. Acad. Sci. U.S.A*. 2016;**113**:10601-10606 https://doi.org/10.1073/pnas.1600359113
5. Ganesh S, Bristow LA, Larsen M *et al.* Size-fraction partitioning of community gene transcription and nitrogen metabolism in a marine oxygen minimum zone. *ISME J*. 2015;**9**:2682-2696 https://doi.org/10.1038/ismej.2015.44
6. Lipschultz F, Wofsy S, Ward B *et al.* Bacterial transformations of inorganic nitrogen in the oxygen-deficient waters of the eastern tropical south pacific ocean. *Deep-Sea Res. I: Oceanogr. Res. Pap.* 1990;**37**:1513-1541 https://doi.org/10.1016/0198-0149(90)90060-9
7. Peng X, Fuchsman CA, Jayakumar A *et al.* Revisiting nitrification in the eastern tropical south pacific: A focus on controls. *J. of Geophys. Res: Oceans*. 2016;**121**:1667-1684 https://doi.org/10.1002/2015JC011455
8. Kalvelage T, Lavik G, Lam P *et al.* Nitrogen cycling driven by organic matter export in the south pacific oxygen minimum zone. *Nat. Geosci*. 2013;**6**:228-234 https://doi.org/10.1038/ngeo1739
9. Minh BQ, Schmidt HA, Chernomor O *et al.* Iq-tree 2: New models and efficient methods for phylogenetic inference in the genomic era. *Mol. Biol. Evol*. 2020;**37**:1530-1534 https://doi.org/10.1093/molbev/msaa015
10. Katoh K, Standley DM. Mafft multiple sequence alignment software version 7: Improvements in performance and usability. *Mol. Biol. and Evol*. 2013;**30**:772-780 https://doi.org/10.1093/molbev/mst010
11. Capella-Gutiérrez S, Silla-Martínez JM, Gabaldón T. Trimal: A tool for automated alignment trimming in large-scale phylogenetic analyses. *Bioinformatics*. 2009;**25**:1972-1973 https://doi.org/10.1093/bioinformatics/btp348
